# Statistical tools for synthesizing lists of differentially expressed features in related experiments

**DOI:** 10.1186/gb-2007-8-4-r54

**Published:** 2007-04-11

**Authors:** Marta Blangiardo, Sylvia Richardson

**Affiliations:** 1 Centre for Biostatistics, Imperial College, St Mary's Campus, Norfolk Place, London W2 1PG, UK

## Abstract

We propose a novel approach for finding a list of features that are commonly perturbed in two or more experiments, quantifying the evidence of dependence between the experiments by a ratio. We present a Bayesian analysis of this ratio, which leads us to suggest two rules for choosing a cut-off on the ranked list of *p *values. We evaluate and compare the performance of these statistical tools in a simulation study, and show their usefulness on two real datasets.

## Abstract 2

A novel approach for finding a list of features that are commonly perturbed in two or more experiments, quantifying the evidence of dependence between the experiments by a ratio.

## Background

In the microarray framework researchers are often interested in the comparison of two or more similar experiments that involve different treatments/exposures, tissues, or species. The aim is to find common denominators between these experiments in the form of a parsimonious list of features (for example, genes, biological processes) for which there is strong evidence that the listed features are commonly perturbed in both (all) the experiments and from which to start further investigations. For example, finding common perturbation of a known pathway in several tissues will indicate that this pathway is involved in a systemic response, which is conserved between tissues.

Ideally, such a problem should involve the joint re-analysis of the two (all) experiments, but this is not always easily feasible (for example, different platforms), and is, in any case, computationally demanding. Alternatively, a natural approach is to consider the ranked list of features derived in each experiment, and to define a process by which a meaningful intersection of the lists can be computed and statistically assessed.

Methods to synthesize probability measures from several experiments (for example, *p *values) have been proposed in the literature. Rhodes *et al*. in 2002 [1] applied Fisher's inverse chi square test to lists of *p *values from different experiments, with the aim of pooling them together in a meta-analysis. The idea has been improved and enlarged by Hwang *et al*. [2], who proposed to assign different weights to different experiments and introduced two more statistics in addition to Fisher's weighted F (Mudholkar-George's weighted T and Liptak-Stouffer's weighted Z). However, as these methods look at evidence of global differential expression across the experiments and define sets of genes based on the global *p *values, their aim is different from ours: we could say that they are focused on statistically assessing the union of different experiments while we are interested in their intersection.

The best statistical approach that aims to evaluate the strength of the intersection remains an open question, as discussed recently by Allison *et al*. [3]. As a first approach, the authors suggest that by using a pre-specified threshold on the *p *value for differential expression in each experiment, the outcomes of two experiments can be treated as two dichotomous variables. A chi-square test of independence can then be performed to evaluate whether the degree of overlap between experiments is greater than expected by chance. But this way of proceeding is heavily dependent on the choice of a threshold used to dichotomize the outcome of the two experiments and neglects useful information on degrees of evidence of differential expression in each experiment.

We propose a novel and powerful method for synthesizing such lists that is based on two ideas. Firstly, the departure from the null hypothesis of a chance association between the results of each experiment is characterized by a ratio measuring the relative increase of the number of features in common with respect to the number expected by chance. Secondly, the statistical significance of the ratio is assessed and exploited to propose rules to define synthesized lists.

For the sake of clarity, from now on we will discuss our methodology in the context of gene expression experiments where the features of interest are genes and the aim is to synthesize lists of differentially expressed genes. But we stress that our methodology is applicable to synthesize ranked lists of any feature of interest from a variety of experiments, as long as each feature is associated with a 'measure of interest' on a probability scale.

Representing the data in a series of 2 × 2 contingency tables, we first specify a (conditional) model of independence that treats the marginal frequencies in each list as fixed quantities: we calculate the ratio between observed and expected number of genes in common for each table and focus attention on the maximum ratio, that is, the strongest deviation from independence. We propose a permutation based test to assess its significance and discuss some shortcomings of this simple approach.

We enlarge the scenario by specifying a joint model of the two experiments (treating the marginal frequencies of differential expression in each experiment as random quantities, instead of fixed) that is formulated in a Bayesian framework. Inference can be based on the marginal posterior distribution of the maximum of the ratio of the observed to the expected probability of genes to be in common.

Note that procedures based on maximum statistics are used in a variety of contexts to focus the analysis on particular subsets of interest; for example, in geographical epidemiology as a way of investigating maximum disease risks around a point source [4], or for scanning time or spatial windows for clusters of cases [5]. In gene expression studies, maximum-based statistics have been proposed for evaluating if *a priori *defined gene sets are enriched relative to a list of genes ranked on the basis of their differential expression between two classes [6].

Focusing on the maximal ratio we are not aiming at finding the largest list of genes in common, but we are interested in a parsimonious list associated with the strongest evidence of dependence between experiments. However, by being very specific (few false positives), this procedure tends to be rather conservative and to be associated with a narrow list of genes in common. To increase sensitivity and account for larger lists, we propose a second rule that focuses attention on the list associated with a ratio equal to or greater than two. We show in our simulations that this rule leads to a good compromise of false positives and false negatives, indicating very high specificity and good sensitivity. It is also close to achieving the minimum of the total error (sum of false positives and false negatives).

We evaluate the performance of our methodology on simulated data and compare the results to those obtained using Hwang *et al*.'s approach. Then, we apply our method to two real case studies, highlighting the biological interest of the obtained results.

## Results

We demonstrate the statistical and biological potential of our methodology using simulated data and publicly available datasets. For the simulation we follow the setup described in [2]. The first real example uses public data from an experiment that evaluates the effect of mechanical ventilation on lung gene expression of mice and rats. The second real example uses public data from an experiment that evaluates the effect of high fat diet on fat and skeletal muscle of mice.

### 2 × 2 Table: conditional model for two experiments

Suppose we want to compare the results of two microarray experiments, each of them reporting for the same set of *n *genes a measure of differential expression on a probability scale (for example, *p *value; Table 1).

**Table 1:** Lists of *p *values for two experiments

Experiment A	Experiment B
pA1 MathType@MTEF@5@5@+=feaafiart1ev1aaatCvAUfeBSjuyZL2yd9gzLbvyNv2Caerbhv2BYDwAHbqedmvETj2BSbqee0evGueE0jxyaibaiKI8=vI8tuQ8FMI8Gi=hEeeu0xXdbba9frFj0=OqFfea0dXdd9vqai=hGuQ8kuc9pgc9s8qqaq=dirpe0xb9q8qiLsFr0=vr0=vr0dc8meaabaqaciGacaGaaeqabaqadeqadaaakeaacaWGWbWaaSbaaSqaaiaadgeadaWgaaadbaGaaGymaaqabaaaleqaaaaa@3607@	pB1 MathType@MTEF@5@5@+=feaafiart1ev1aaatCvAUfeBSjuyZL2yd9gzLbvyNv2Caerbhv2BYDwAHbqedmvETj2BSbqee0evGueE0jxyaibaiKI8=vI8tuQ8FMI8Gi=hEeeu0xXdbba9frFj0=OqFfea0dXdd9vqai=hGuQ8kuc9pgc9s8qqaq=dirpe0xb9q8qiLsFr0=vr0=vr0dc8meaabaqaciGacaGaaeqabaqadeqadaaakeaacaWGWbWaaSbaaSqaaiaadkeadaWgaaadbaGaaGymaaqabaaaleqaaaaa@3608@
pA2 MathType@MTEF@5@5@+=feaafiart1ev1aaatCvAUfeBSjuyZL2yd9gzLbvyNv2Caerbhv2BYDwAHbqedmvETj2BSbqee0evGueE0jxyaibaiKI8=vI8tuQ8FMI8Gi=hEeeu0xXdbba9frFj0=OqFfea0dXdd9vqai=hGuQ8kuc9pgc9s8qqaq=dirpe0xb9q8qiLsFr0=vr0=vr0dc8meaabaqaciGacaGaaeqabaqadeqadaaakeaacaWGWbWaaSbaaSqaaiaadgeadaWgaaadbaGaaGOmaaqabaaaleqaaaaa@3608@	pB2 MathType@MTEF@5@5@+=feaafiart1ev1aaatCvAUfeBSjuyZL2yd9gzLbvyNv2Caerbhv2BYDwAHbqedmvETj2BSbqee0evGueE0jxyaibaiKI8=vI8tuQ8FMI8Gi=hEeeu0xXdbba9frFj0=OqFfea0dXdd9vqai=hGuQ8kuc9pgc9s8qqaq=dirpe0xb9q8qiLsFr0=vr0=vr0dc8meaabaqaciGacaGaaeqabaqadeqadaaakeaacaWGWbWaaSbaaSqaaiaadkeadaWgaaadbaGaaGOmaaqabaaaleqaaaaa@3609@
...	...
pAn MathType@MTEF@5@5@+=feaafiart1ev1aaatCvAUfeBSjuyZL2yd9gzLbvyNv2Caerbhv2BYDwAHbqedmvETj2BSbqee0evGueE0jxyaibaiKI8=vI8tuQ8FMI8Gi=hEeeu0xXdbba9frFj0=OqFfea0dXdd9vqai=hGuQ8kuc9pgc9s8qqaq=dirpe0xb9q8qiLsFr0=vr0=vr0dc8meaabaqaciGacaGaaeqabaqadeqadaaakeaacaWGWbWaaSbaaSqaaiaadgeadaWgaaadbaGaamOBaaqabaaaleqaaaaa@363F@	pBn MathType@MTEF@5@5@+=feaafiart1ev1aaatCvAUfeBSjuyZL2yd9gzLbvyNv2Caerbhv2BYDwAHbqedmvETj2BSbqee0evGueE0jxyaibaiKI8=vI8tuQ8FMI8Gi=hEeeu0xXdbba9frFj0=OqFfea0dXdd9vqai=hGuQ8kuc9pgc9s8qqaq=dirpe0xb9q8qiLsFr0=vr0=vr0dc8meaabaqaciGacaGaaeqabaqadeqadaaakeaacaWGWbWaaSbaaSqaaiaadkeadaWgaaadbaGaamOBaaqabaaaleqaaaaa@3640@

We rank the genes according to the recorded probability measures. For each cut-off *q*,(0 ≤ *q *≤ 1), we obtain the number of differentially expressed genes for each of the two lists as *O*_1+_(*q*) and *O*_+1_(*q*) and the number *O*_11_(*q*) of differentially expressed genes in common between the two experiments (Table 2). The threshold *q *is a continuous variable but, in practice, we consider a discretization of *q*. In the present paper, we specify a vector *q *= (*q*_0 _= 0, *q*_1 _= 0.001. ..., *q*, ..., *q*_*k *_= 1), formed by *K *= 101 elements, but other discretizations can be used without loss of generality. For a threshold *q*, under the hypothesis of independence of the contrasts investigated by the two experiments, the number of genes in common by chance is calculated as:

**Table 2:** Contingency table for experiment A and experiment B, given a threshold *q*

		Experiment B		
			
		*DE*	*Non DE*	
Experiment A	*DE*	*O*_11_(*q*)	*O*_1+_(*q*) - *O*_11_(*q*)	*O*_1+_(*q*)
	*Non DE*	*O*_+1_(*q*) - *O*_11_(*q*)	*n *- *O*_1+_(*q*) - *O*_+1_(*q*) + *O*_11_(*q*)	*n *- *O*_1+_(*q*)
		*O*_+1_(*q*)	*n *- *O*_+1_(*q*)	*n*

*n *is the total number of genes and *O*_11_(*q*) is the number of genes in common. DE, differentially expressed. Non DE, non differentially expressed

O1+(q)×O+1(q)n
 MathType@MTEF@5@5@+=feaafiart1ev1aaatCvAUfeBSjuyZL2yd9gzLbvyNv2Caerbhv2BYDwAHbqedmvETj2BSbqee0evGueE0jxyaibaiKI8=vI8tuQ8FMI8Gi=hEeeu0xXdbba9frFj0=OqFfea0dXdd9vqai=hGuQ8kuc9pgc9s8qqaq=dirpe0xb9q8qiLsFr0=vr0=vr0dc8meaabaqaciGacaGaaeqabaqadeqadaaakeaadaWcaaqaaiaad+eadaWgaaWcbaGaaGymaiabgUcaRaqabaGccaGGOaGaamyCaiaacMcacqGHxdaTcaWGpbWaaSbaaSqaaiabgUcaRiaaigdaaeqaaOGaaiikaiaadghacaGGPaaabaGaamOBaaaaaaa@4033@

In the 2 × 2 Table, where the marginal frequencies *O*_1+_(*q*), *O*_+1_(*q*) and the total number of genes *n *are assumed fixed quantities, given *q*, the only random variable is *O*_11_(*q*).

The conditional distribution of *O*_11_(*q*) is hypergeometric [7]:

*O*_11_(*q*) ~ *Hyper*(*O*_1+_(*q*), *O*_+1_(*q*), *n*).

We then calculate the statistic *T*(*q*) as the observed to expected ratio:

T(q)=O11(q)O1+(q)×O+1(q)n.
 MathType@MTEF@5@5@+=feaafiart1ev1aaatCvAUfeBSjuyZL2yd9gzLbvyNv2Caerbhv2BYDwAHbqedmvETj2BSbqee0evGueE0jxyaibaiKI8=vI8tuQ8FMI8Gi=hEeeu0xXdbba9frFj0=OqFfea0dXdd9vqai=hGuQ8kuc9pgc9s8qqaq=dirpe0xb9q8qiLsFr0=vr0=vr0dc8meaabaqaciGacaGaaeqabaqadeqadaaakeaacaWGubGaaiikaiaadghacaGGPaGaeyypa0ZaaSaaaeaacaWGpbWaaSbaaSqaaiaaigdacaaIXaaabeaakiaacIcacaWGXbGaaiykaaqaamaalaaabaGaam4tamaaBaaaleaacaaIXaGaey4kaScabeaakiaacIcacaWGXbGaaiykaiabgEna0kaad+eadaWgaaWcbaGaey4kaSIaaGymaaqabaGccaGGOaGaamyCaiaacMcaaeaacaWGUbaaaaaacaGGUaaaaa@49F2@

In other words, *T*(*q*) quantifies the strength of association between lists at cut-off *q *in terms of ratio of observed to expected. The denominator is a fixed quantity, so the distribution of *T*(*q*) is also proportional to a hypergeometric distribution:

*T*_q _∝ *Hyper*(*O*_1+_(*q*), *O*_+1_(*q*), *n*)

with mean and variance:

*E*(*T*(*q*)|*O*_1+_(*q*), *O*_+1_(*q*), *n*) = 1

Var(T(q)|O1+(q),O+1(q),n)=(1−O1+(q)n)×(n−O+1(q)n−1).
 MathType@MTEF@5@5@+=feaafiart1ev1aaatCvAUfeBSjuyZL2yd9gzLbvyNv2Caerbhv2BYDwAHbqedmvETj2BSbqee0evGueE0jxyaibaiKI8=vI8tuQ8FMI8Gi=hEeeu0xXdbba9frFj0=OqFfea0dXdd9vqai=hGuQ8kuc9pgc9s8qqaq=dirpe0xb9q8qiLsFr0=vr0=vr0dc8meaabaqaciGacaGaaeqabaqadeqadaaakeaacaWGwbGaamyyaiaadkhacaGGOaGaamivaiaacIcacaWGXbGaaiykaiaacYhacaWGpbWaaSbaaSqaaiaaigdacqGHRaWkaeqaaOGaaiikaiaadghacaGGPaGaaiilaiaad+eadaWgaaWcbaGaey4kaSIaaGymaaqabaGccaGGOaGaamyCaiaacMcacaGGSaGaamOBaiaacMcacqGH9aqpdaqadaqaaiaaigdacqGHsisldaWcaaqaaiaad+eadaWgaaWcbaGaaGymaiabgUcaRaqabaGccaGGOaGaamyCaiaacMcaaeaacaWGUbaaaaGaayjkaiaawMcaaiabgEna0oaabmaabaWaaSaaaeaacaWGUbGaeyOeI0Iaam4tamaaBaaaleaacqGHRaWkcaaIXaaabeaakiaacIcacaWGXbGaaiykaaqaaiaad6gacqGHsislcaaIXaaaaaGaayjkaiaawMcaaiaac6caaaa@5FA8@

Throughout, we use the symbol | to denote conditioning, thus *E*(*T*(*q*)|*O*_1+_(*q*), *O*_+1_(*q*), *n*) indicates the conditional expectation of *T*(*q*) given *O*_1+_(*q*), *O*_+1_(*q*) and *n*.

As a first step, we focus attention on the ordinal statistic *T*(*q*_*max*_) ≡ *max*_*q*_*T*(*q*), which represents the maximal deviation from the null model of independence between the two experiments, or equivalently the largest relative increase of the number of genes in common. This maximum value is associated with a threshold *q*_max _on the probability measure and with a number *O*_11_(*q*_max_) of genes in common, which can be selected for further investigations and mined for relevant biological pathways.

The exact distribution of *T*(*q*_max_) is not easily obtained, since the series of 2 × 2 tables are not independent. We thus suggest performing a Monte Carlo permutation test of *T*(*q*) under the null hypothesis of independence between the two experiments. To be precise, the probability measures of one list are randomly permuted *S *times, while those of the other list are kept fixed, leading to *S *values of the statistic *T*^*S*^(*q*_*max*_), which represent the null distribution of *T*(*q*_max_). From these, a Monte Carlo *p *value for the observed value of *T*(*q*_max_) can be computed and the choice of *S *adapted to the required degree of precision.

### 2 × 2 Table: joint model of two experiments

For extreme values of the threshold *q *(*q *≅ 0), *O*_1+_(*q*) and *O*_+1_(*q*) can be very small. In this case, the denominator of *T*(*q*) assumes values smaller than 1 and *T*(*q*) explodes, leading to unreliable estimates of the ratio. In addition, the hypergeometric sampling model specified for *T*(*q*_max_) in our previous procedure does not take into account the uncertainty of the margins of the table (since they are all considered fixed).

To address these issues and to improve our statistical procedure, we thus propose to consider a joint model of the experiments, which also treats *O*_1+_(*q*) and *O*_+1_(*q*) as random variables, releasing the conditioning. Furthermore, we specify this in a Bayesian framework, where the underlying probabilities,

θi(q),1≤i≤4,∑i=14θi(q)=1,
 MathType@MTEF@5@5@+=feaafiart1ev1aaatCvAUfeBSjuyZL2yd9gzLbvyNv2Caerbhv2BYDwAHbqedmvETj2BSbqee0evGueE0jxyaibaiKI8=vI8tuQ8FMI8Gi=hEeeu0xXdbba9frFj0=OqFfea0dXdd9vqai=hGuQ8kuc9pgc9s8qqaq=dirpe0xb9q8qiLsFr0=vr0=vr0dc8meaabaqaciGacaGaaeqabaqadeqadaaakeaaiiGacqWF4oqCdaWgaaWcbaGaamyAaaqabaGccaGGOaGaamyCaiaacMcacaGGSaGaaGymaiabgsMiJkaadMgacqGHKjYOcaaI0aGaaiilamaaqahabaGae8hUde3aaSbaaSqaaiaadMgaaeqaaOGaaiikaiaadghacaGGPaaaleaacaWGPbGaeyypa0JaaGymaaqaaiaaisdaa0GaeyyeIuoakiabg2da9iaaigdacaGGSaaaaa@4CDC@

for the four cells in the 2 × 2 contingency table (indexes from left to right) are given a prior distribution. In this way, we account for the variability in *O*_1+_(*q*) and *O*_+1_(*q*) and smooth the ratio *T*(*q*) for extreme, small values of *q*.

Starting from Table 2, we model the observed frequencies as arising from a multinomial distribution:

Multi(O|θ,n)αθ1(q)O11(q)×θ2(q)[O1+(q)−O11(q)]×θ3(q)[O+1(q)−O11(q)]×θ4(q)[n−O1+(q)−O+1(q)+O11(q)]
 MathType@MTEF@5@5@+=feaafiart1ev1aaatCvAUfeBSjuyZL2yd9gzLbvyNv2Caerbhv2BYDwAHbqedmvETj2BSbqee0evGueE0jxyaibaiKI8=vI8tuQ8FMI8Gi=hEeeu0xXdbba9frFj0=OqFfea0dXdd9vqai=hGuQ8kuc9pgc9s8qqaq=dirpe0xb9q8qiLsFr0=vr0=vr0dc8meaabaqaciGacaGaaeqabaqadeqadaaakeaacaWGnbGaamyDaiaadYgacaWG0bGaamyAaiaacIcaieqacaWFpbGaaiiFaGGaciab+H7aXjaacYcacaWGUbGaaiykaiab+f7aHjab+H7aXnaaBaaaleaacaaIXaaabeaakiaacIcacaWGXbGaaiykamaaCaaaleqabaGaam4tamaaBaaameaacaaIXaGaaGymaaqabaWccaGGOaGaamyCaiaacMcaaaGccqGHxdaTcqGF4oqCdaWgaaWcbaGaaGOmaaqabaGccaGGOaGaamyCaiaacMcadaahaaWcbeqaaiaacUfacaWGpbWaaSbaaWqaaiaaigdacqGHRaWkaeqaaSGaaiikaiaadghacaGGPaGaeyOeI0Iaam4tamaaBaaameaacaaIXaGaaGymaaqabaWccaGGOaGaamyCaiaacMcacaGGDbaaaOGaey41aqRae4hUde3aaSbaaSqaaiaaiodaaeqaaOGaaiikaiaadghacaGGPaWaaWbaaSqabeaacaGGBbGaam4tamaaBaaameaacqGHRaWkcaaIXaaabeaaliaacIcacaWGXbGaaiykaiabgkHiTiaad+eadaWgaaadbaGaaGymaiaaigdaaeqaaSGaaiikaiaadghacaGGPaGaaiyxaaaakiabgEna0kab+H7aXnaaBaaaleaacaaI0aaabeaakiaacIcacaWGXbGaaiykamaaCaaaleqabaGaai4waiaad6gacqGHsislcaWGpbWaaSbaaWqaaiaaigdacqGHRaWkaeqaaSGaaiikaiaadghacaGGPaGaeyOeI0Iaam4tamaaBaaameaacqGHRaWkcaaIXaaabeaaliaacIcacaWGXbGaaiykaiabgUcaRiaad+eadaWgaaadbaGaaGymaiaaigdaaeqaaSGaaiikaiaadghacaGGPaGaaiyxaaaaaaa@8CC6@

Since we are in a Bayesian framework, we need to specify a prior distribution for all the parameters. The vector of parameters *θ*(*q*) is modeled as arising from a Dirichlet distribution [8]:

*θ*(*q*) ~ *Dir*(*a*, *a*, *a*, *a*), *a *= 0.05,

which ensures the constraint ∑i=14θi(q)=1
 MathType@MTEF@5@5@+=feaafiart1ev1aaatCvAUfeBSjuyZL2yd9gzLbvyNv2Caerbhv2BYDwAHbqedmvETj2BSbqee0evGueE0jxyaibaiKI8=vI8tuQ8FMI8Gi=hEeeu0xXdbba9frFj0=OqFfea0dXdd9vqai=hGuQ8kuc9pgc9s8qqaq=dirpe0xb9q8qiLsFr0=vr0=vr0dc8meaabaqaciGacaGaaeqabaqadeqadaaakeaadaaeWaqaaGGaciab=H7aXnaaBaaaleaacaWGPbaabeaakiaacIcacaWGXbGaaiykaiabg2da9iaaigdaaSqaaiaadMgacqGH9aqpcaaIXaaabaGaaGinaaqdcqGHris5aaaa@3F8D@.

The derived quantity of interest is, as before, the ratio of the probability that a differentially expressed gene is truly common for both experiments, to the probability that a gene is included in the common list by chance:

R(q)=θ1(q)(θ1(q)+θ2(q))×(θ1(q)+θ3(q)).
 MathType@MTEF@5@5@+=feaafiart1ev1aaatCvAUfeBSjuyZL2yd9gzLbvyNv2Caerbhv2BYDwAHbqedmvETj2BSbqee0evGueE0jxyaibaiKI8=vI8tuQ8FMI8Gi=hEeeu0xXdbba9frFj0=OqFfea0dXdd9vqai=hGuQ8kuc9pgc9s8qqaq=dirpe0xb9q8qiLsFr0=vr0=vr0dc8meaabaqaciGacaGaaeqabaqadeqadaaakeaacaWGsbGaaiikaiaadghacaGGPaGaeyypa0ZaaSaaaeaacqaH4oqCdaWgaaWcbaGaaGymaaqabaGccaGGOaGaamyCaiaacMcaaeaacaGGOaGaeqiUde3aaSbaaSqaaiaaigdaaeqaaOGaaiikaiaadghacaGGPaGaey4kaSIaeqiUde3aaSbaaSqaaiaaikdaaeqaaOGaaiikaiaadghacaGGPaGaaiykaiabgEna0kaacIcacqaH4oqCdaWgaaWcbaGaaGymaaqabaGccaGGOaGaamyCaiaacMcacqGHRaWkcqaH4oqCdaWgaaWcbaGaaG4maaqabaGccaGGOaGaamyCaiaacMcacaGGPaaaaiaac6caaaa@5779@

The Dirichlet prior is conjugate for the multinomial likelihood [8] and the posterior distribution of *θ*(*q*)|***O***, *n *is again a Dirichlet distribution, given by:

θ|O,n~Dir(O11(q)+a,[O1+(q)−O11(q)]+a,[O+1(q)−O11(q)]+a,[n−O1+(q)−O+1(q)+O11(q)]+a)
 MathType@MTEF@5@5@+=feaafiart1ev1aaatCvAUfeBSjuyZL2yd9gzLbvyNv2Caerbhv2BYDwAHbqedmvETj2BSbqee0evGueE0jxyaibaiKI8=vI8tuQ8FMI8Gi=hEeeu0xXdbba9frFj0=OqFfea0dXdd9vqai=hGuQ8kuc9pgc9s8qqaq=dirpe0xb9q8qiLsFr0=vr0=vr0dc8meaabaqaciGacaGaaeqabaqadeqadaaakeaacqaH4oqCcaGG8bacbeGaa83taiaacYcacaWGUbGaaiOFaiaadseacaWGPbGaamOCaiaacIcacaWGpbWaaSbaaSqaaiaaigdacaaIXaaabeaakiaacIcacaWGXbGaaiykaiabgUcaRiaadggacaGGSaGaai4waiaad+eadaWgaaWcbaGaaGymaiabgUcaRaqabaGccaGGOaGaamyCaiaacMcacqGHsislcaWGpbWaaSbaaSqaaiaaigdacaaIXaaabeaakiaacIcacaWGXbGaaiykaiaac2facqGHRaWkcaWGHbGaaiilaiaacUfacaWGpbWaaSbaaSqaaiabgUcaRiaaigdaaeqaaOGaaiikaiaadghacaGGPaGaeyOeI0Iaam4tamaaBaaaleaacaaIXaGaaGymaaqabaGccaGGOaGaamyCaiaacMcacaGGDbGaey4kaSIaamyyaiaacYcacaGGBbGaamOBaiabgkHiTiaad+eadaWgaaWcbaGaaGymaiabgUcaRaqabaGccaGGOaGaamyCaiaacMcacqGHsislcaWGpbWaaSbaaSqaaiabgUcaRiaaigdaaeqaaOGaaiikaiaadghacaGGPaGaey4kaSIaam4tamaaBaaaleaacaaIXaGaaGymaaqabaGccaGGOaGaamyCaiaacMcacaGGDbGaey4kaSIaamyyaiaacMcaaaa@7876@

This distribution is easily sampled from using standard algorithms. Note that the prior weights *a *= 0.05 can be interpreted as the number of hypothetical counts in each cell observed prior to the investigation. Further, it can be shown that the variance of the vector of probabilities in the Dirichlet distribution increases as the prior weights tend to zero. Thus, our choice of value of 0.05 for the prior weights allows both high variability and a small influence of the prior specification on the posterior distribution of *θ*(*q*). The posterior distribution of *R*(*q*)|***O***, *n *can be easily derived from that of *θ*(*q*) using for example a sample of values of *θ*(*q*), generated from the posterior distribution (equation 5). In particular, from a sample of values of *R*(*q*)|***O***, *n*, the 95% two sided credibility interval, CI_95_(q), can be easily computed, for each *R*(*q*).

### 2 × 2 Table: decision rules for intersection

**Figure 1:**
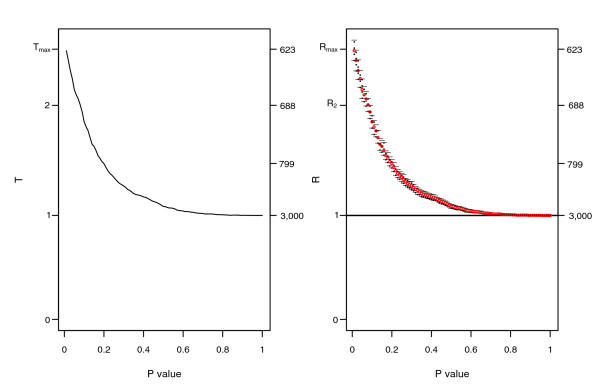
Typical plots of *T*(*q*) and *R*(*q*) for associated experiments (case A1). The two associated experiments were simulated under scenario I, structure A, with true differences drawn from a *Ga*(2.5,0.4) and noise experiment specific of 0.5 and 0.8, respectively (signal-to-noise ratio = 9.6). The left plot shows the distribution of *T*(*q*) and the right one shows the distribution of *R*(*q*) with Bayesian credibility intervals at 95%. *T*(*q*) shows a deviation from 1 for a *p *value between 0.01 and 0.5. T(q_max_) is 2.6 and corresponds to a threshold *q *= 0.01. *R*(*q*) presents the same trend, but the estimates are slightly smaller since the model takes into account the variability of the margins of the 2 × 2 table. The threshold associated with *R*(*q*) = 2 is 0.08. The number of genes in common for each ratio *R*(*q*) is reported on the right axis of each plot.

In the Bayesian context, several decision rules can be envisaged to choose the threshold corresponding to the common list showing a clear evidence of association between experiments. The general principle is as follows: first, select a ratio *R*(*q*) according to a decision rule; second, consider the threshold *q *corresponding to the selected ratio; and third, return the list *O*_11_(*q*), that is, the intersection of the lists for the threshold *q*. Figure 1 (right) shows a typical plot of *R*(*q*) and its credibility interval as a function of *q *in case of associated experiments (a different shape for *R*(*q*) is presented in Additional data file 1). As the *p *value increases, the ratio *R*(*q*) decreases and the associated list of common genes *O*_11_(*q*) becomes larger (the number of genes in common for each ratio is indicated on the right axis of the plot). We need a rule to select a threshold on the *p *value and the corresponding list of genes in common. To this purpose we now discuss two decision rules.

Under the null model of no association between the experiments, *Median*(*R*(*q*)|*H*_0_) = 1, so we consider *R*(*q*) as indicating departure from independence if its credibility interval does not contain 1.

As an extension of *T*(*q*_max_) we thus propose to consider the maximum of *Median*(*R*(*q*)|***O***, *n*) only for the subset of credibility intervals that do not include 1 and define:

q_max _= argmax{*Median*(*R*(*q*)|***O***, *n*) over the set of values of *q *for which *CI*_95_(*q*) excludes 1}.

In other words, *q*_max _is defined to be the threshold associated with the maximum of the ratio, which we denote *R*(*q*_max_). If all credibility intervals contain 1, the maximum of *R*(*q*) can still be computed, but we do not associate it with a list since there is no departure from independence that could be considered significant.

Note that in the Bayesian context many *R*(*q*) can have a CI that excludes 1 and they all represent a significant deviation from the independence. An advantage of the maximum statistic is that it returns a list of interesting features with few false positives (FP), as will be shown later in the simulations. On the other hand, this list is usually rather small and in cases where the level of noise is substantial it excludes a large number of true positives (TP), for which the evidence is less strong.

We next consider an alternative to the max ratio: the largest threshold *q *for which the ratio *R*(*q*) ≥ 2. It is the largest threshold where the number of genes called in common at least doubles the number of genes in common under independence:

*q*_2 _= max{over the set of values of *q *for which *Median*(*R*(*q*)|***O***, *n*) ≥ 2 and *CI*_95_(*q*) excludes 1}.

Using this rule provides a fair balance between specificity and sensitivity as we will show later. Indeed, it is expected that when going beyond this point to larger values of *q*, the marginal benefit of adding a few more true positives and of reducing the false negatives (FN) to the list will be outweighed by the expected larger number of false positives that would also be added. By our simulations we show indeed that this rule is close to giving the minimal global error (FP + FN).

**Figure 2:**
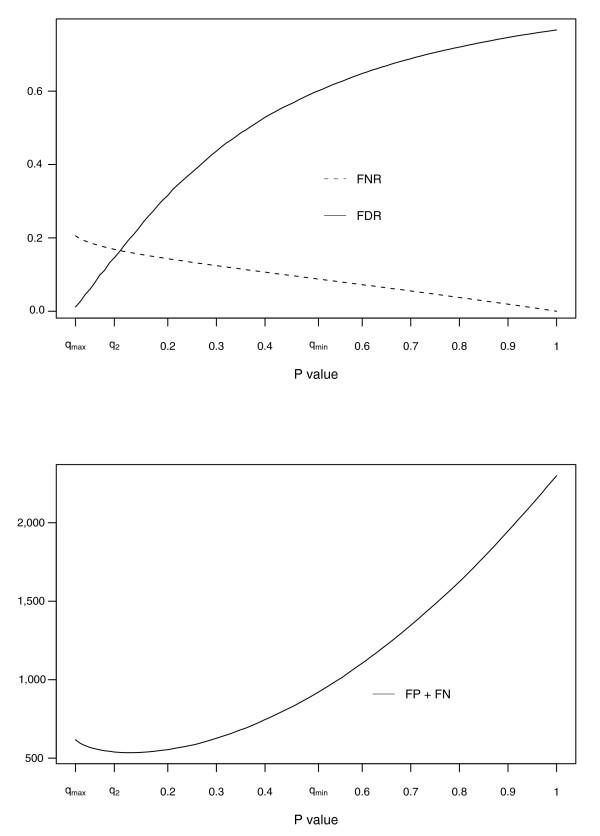
Misclassification error, false discovery and false non-discovery rates for case A2 (results are averaged over 50 replicates). The upper plot shows the false discovery rate (FDR) and the false non-discovery rate (FNR) for case A2. The FDR is calculated as the ratio of the false positives to the number of genes called in common, while the FDR is calculated as the ratio of the false negatives to the number of genes not called in common. The true differences *d*_*g *_are drawn from a *Ga*(2, 0.5) and the noise component experiment specific is 2 for the first experiment and 3 for the second. R(q_max_) shows the minimum FDR. On the other hand, R(q_min_) has a very large FDR and the improvement of the FNR is slight. As a compromise, the threshold q_2 _is close to q_max_, so guarantees a low FDR, but returns a larger list. It approximatively corresponds to the intersection point between the two curves of FDR and FNR. The lower plot shows the global error as the sum of FP and FN. The threshold associated with R(q_2_) is very close to the minimum of the curve, that is, to the smallest global misclassification error.

Figure 2 (top) plots the false discovery rate:

FDR = *FP*(*q*)/*O*_11_(*q*)

and false non-discovery rate:

FNR = *FN*(*q*)/(*n *- *O*_11_(*q*))

for 50 simulations carried out as described in Materials and methods, for scenario I structure A. It is clear that *R*(*q*_max_) has the smallest FDR. On the other hand, *q*_2 _corresponds to the intersection between FDR and FNR. Moreover, in Figure 2 (bottom) we show that the same threshold minimizes the global misclassification error as the sum of false positives and false negatives. Note that if we considered the minimum significant ratio, defined as the minimum of the *R*(*q*) over the set of credibility intervals excluding 1, FDR would increase dramatically and the FNR would decrease only marginally with respect to *R*(*q*_max_) and *R*(*q*_2_). As expected, the global misclassification error would also be much larger, making this rule inappropriate.

When there are no ratios *R*(*q*) equal or greater than 2 (which can happen in the case of large noise or when there is only a small proportion of genes in common), this rule does not apply and we recommend using the rule corresponding to *R*(*q*_max_).

Our computations have been implemented in the statistical programming language R [9]. The R package for simulating the data, for the two tests and for visualizing the results is called BGcom and is available on our project BGX website [10].

### Performance on simulated data

Besides assessing the operating characteristics of our proposed rules, we also applied the method proposed by Hwang *et al*. implemented in Matlab [11]. Note that their aim is to integrate *p *values from different experiments in a meta-analysis and they present three statistics to do so: Fisher's weighted F, Mudholkar-George's weighted T and Liptak-Stouffer's weighted Z. We report Fisher's weighted F (the default statistic in the Matlab function), defined as:

Fg=−2∑k=12wkln(pgk)
 MathType@MTEF@5@5@+=feaafiart1ev1aaatCvAUfeBSjuyZL2yd9gzLbvyNv2Caerbhv2BYDwAHbqedmvETj2BSbqee0evGueE0jxyaibaiKI8=vI8tuQ8FMI8Gi=hEeeu0xXdbba9frFj0=OqFfea0dXdd9vqai=hGuQ8kuc9pgc9s8qqaq=dirpe0xb9q8qiLsFr0=vr0=vr0dc8meaabaqaciGacaGaaeqabaqadeqadaaakeaacaWGgbWaaSbaaSqaaiaadEgaaeqaaOGaeyypa0JaeyOeI0IaaGOmamaaqadabaGaam4DamaaBaaaleaacaWGRbaabeaaieGakiaa=XgacaWFUbGaaiikaiaadchadaWgaaWcbaGaam4zaiaadUgaaeqaaOGaaiykaaWcbaGaam4Aaiabg2da9iaaigdaaeaacaaIYaaaniabggHiLdaaaa@45A1@

where *w*_*k *_is the weight for the *k*^*th *^experiment and *p*_*gk *_is the *p *value for the gene *g *in the experiment *k*. *F*_*g *_will be a new global *p *value that integrates those weights from different experiments. The authors also present several rules to select differentially expressed genes from *F*_*g*_, the simplest one using a fixed threshold on the *p *values equal to 0.05, and others that minimize the number of false positives and false negatives, in a parametric or non-parametric framework. We follow the authors' suggestion and use the non-parametric rule. For more details on the method, see [2].

**Figure 3:**
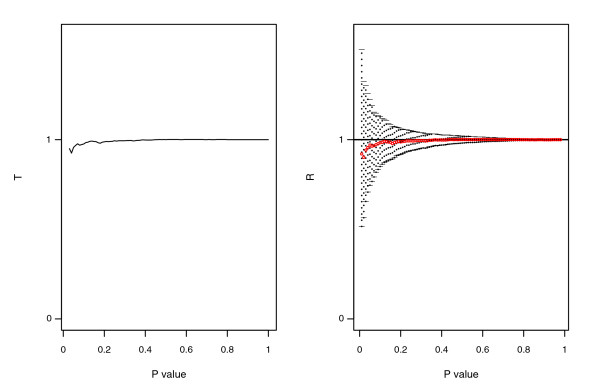
Typical plots of *T*(*q*) and *R*(*q*) in the case of independent experiments. The two independent experiments are simulated under scenario I, structure A, with true differences drawn from a *Ga*(1, 1) and noise experiment specific of 2 and 2.5, respectively (signal-to-noise ratio = 0.4). The left plot shows the distribution of *T*(*q*) and the right one shows the distribution of *R*(*q*) with Bayesian credibility intervals at 95%. *T*(*q*) follows a horizontal line of height 1 (independence between the lists) and presents instability for small *p *values (left tail). The Bayesian model does not present any significant threshold for which *R*(*q*) deviates from 1 and the CI_95 _always includes 1.

The behavior of *T*(*q*) and of the credibility intervals *CI*_95_(*q*) for a typical simulation are displayed in Figure 1 (associated experiments) and Figure 3 (independent experiments). When the two experiments are not associated (the number of simulated genes in common is equal to 0), the plot of *T*(*q*) for different cut-offs *q *is, as expected, a horizontal line of height 1, with evidence of noise for small *p *values. In the same Figure, one sees that all the credibility intervals derived by the Bayesian procedure include the value 1 and have decreasing width as *q *gets larger, as expected.

In the case of two independent experiments we never declare any gene to be in common in any of the 50 simulations, so our procedure has no error. On the other hand, Hwang *et al*.'s method picks up 320 genes on average (Table 3, independence case), which are all false positives.

**Table 3:** Performance of Hwang *et al*.'s method on simulated data for scenario I

	DE	nonDE	FP (%)	TP (%)	FN (%)	TN (%)	Global error	Global error *R*(*q*_2_)
**Independent case: *n *= 3000, common = 0, DE1 = 1000, DE2 = 800**	320	2,680	320 (10.7)	0	0	2,680 (89.3)	320	0
								
**A: *n *= 3000, common = 700, DE1 = 1000, DE2 = 800**								
Case A1	1,121	1,879	440 (19.1)	681 (97.3)	19 (2.7)	1,860 (80.9)	459	82
Case A2	409	2,591	188 (8.2)	221 (31.6)	479 (68.4)	2,112 (91.8)	667	544
								
**B: *n *= 3000, common = 200, DE1 = 700, DE2 = 500**								
Case B1	999	2,001	805 (28.8)	194 (97.0)	6 (3.0)	1,996 (71.2)	811	31*
Case B2	427	2,573	333 (11.9)	94 (47.0)	106 (53.0)	2,467 (88.1)	439	165
								
**C: *n *= 3000, common = 100, DE1 = 500, DE2 = 400**								
Case C1	816	2,185	718 (24.8)	97 (97.1)	3 (2.9)	2,182 (75.2)	721	19*
Case C2	346	2,654	299 (10.3)	47 (47.0)	53 (53.0)	2,601 (89.7)	352	84

Average simulation results: we present the results from Hwang *et al*.'s method on the simulated data under scenario I. DE1 and DE2 are the differentially expressed genes in the first and the second experiment respectively. We used the Fisher's weighted F defined as Fg=−2∑k=12wkln(pgk)
 MathType@MTEF@5@5@+=feaafiart1ev1aaatCvAUfeBSjuyZL2yd9gzLbvyNv2Caerbhv2BYDwAHbqedmvETj2BSbqee0evGueE0jxyaibaiKI8=vI8tuQ8FMI8Gi=hEeeu0xXdbba9frFj0=OqFfea0dXdd9vqai=hGuQ8kuc9pgc9s8qqaq=dirpe0xb9q8qiLsFr0=vr0=vr0dc8meaabaqaciGacaGaaeqabaqadeqadaaakeaacaWGgbWaaSbaaSqaaiaadEgaaeqaaOGaeyypa0JaeyOeI0IaaGOmamaaqadabaGaam4DamaaBaaaleaacaWGRbaabeaaieGakiaa=XgacaWFUbGaaiikaiaadchadaWgaaWcbaGaam4zaiaadUgaaeqaaOGaaiykaaWcbaGaam4Aaiabg2da9iaaigdaaeaacaaIYaaaniabggHiLdaaaa@45A1@, where *w*_*k *_is the weight for the *k*^*th *^experiment and *p*_*gk *_is the *p *value for the gene *g *in the experiment *k*. We present the non-parametric rule to select the differentially expressed (DE) genes, as suggested by the authors. The method is implemented in Matlab. In the last column we report the Global error (FP + FN) of our procedure for *q*_2 _(see Table 2) for ease of comparison. *There is no ratio larger than 2 so the maximum rule has been used in this case.

When there is a positive association between the two experiments, *T*(*q*) can assume two shapes: it can decrease monotonically as the *p *values increase (Figure 1), or reach a peak and then decrease (Additional data file 1) as the *p *values increase. The Bayesian estimates exhibit a similar shape, but since in this approach the variability of the denominator of *T*(*q*) is modeled, the resulting ratio estimates are smoothed.

We see that our proposed method gives a sensible and interpretable procedure, with a pattern that is easily distinguishable from that of the no association case. This is confirmed by the results given in Table 4.

**Table 4:** Performance on simulated data for scenario I

Parameters	Rules	q	R	*CI*_95_	*O*_11_	*O*_1+_	*O*_+1_	FP (%)	TP (%)	FN (%)	TN (%)	Global error
**Independence case: *n *= 3000, common = 0, DE1 = 1000, DE2 = 800**												
Independence: signal to noise		0.55	1*	0.98-1.02	0^†^	0^†^	0^†^	0	0	0	3,000 (100.0)	0
ratio = 0.4^‡^												
												
**A: *n *= 3000, common = 700, DE1 = 1000, DE2 = 800**												
Case A1: signal to noise ratio = 9.6^‡^	Max	0.01	2.60	2.50-2.72	619	975	730	4 (0.2)	615 (87.8)	85 (12.2)	2,296 (99.8)	89
	Double	0.06	2.04	1.97-2.19	676	1,095	877	29 (1.3)	647 (92.4)	53 (7.6)	2,271 (98.7)	82
												*Min*^§ ^= *81*
Case A2: signal to noise ratio = 1.6^‡^	Max	0.01	4.72	4.19-5.29	86	346	157	1 (0.0)	85 (12.1)	615 (87.9)	2,299 (100.0)	616
	Double	0.08	2.01	1.90-2.20	212	677	459	28 (1.2)	184 (26.3)	516 (73.7)	2,272 (98.8)	544
												*Min*^§ ^= *535*
												
**B: *n *= 3000, common = 200, DE1 = 700, DE2 = 500**												
Case B1: signal to noise ratio = 9.6^‡^	Max^¶^	0.01	1.72	1.58-1.86	185	691	467	8 (0.3)	177 (88.5)	23 (11.5)	2,792 (99.7)	31
												*Min*^§ ^= *31*
Case B2: signal to noise ratio = 1.6^‡^	Max	0.01	2.98	2.38-3.71	36	250	145	3 (0.1)	33 (16.7)	167 (83.3)	2,797 (99.9)	170
	Double	0.03	2.03	1.67-2.40	57	355	236	11 (0.4)	46 (23.0)	154 (77.1)	2,789 (99.6)	165
												*Min*^§ ^= *165*
												
**C: *n *= 3000, common = 100, DE1 = 500, DE2 = 400**												
Case C1: signal to noise ratio = 9.6^‡^	Max^¶^	0.01	1.48	1.30-1.67	95	500	383	7 (0.2)	88 (88.4)	12 (11.6)	2,893 (99.8)	19
												*Min*^§ ^= *19*
Case C2: signal to noise ratio = 1.6^‡^	Max	0.01	2.93	2.16-3.83	20	214	96	3 (0.1)	17 (16.6)	83 (83.4)	2,897 (99.9)	86
	Double	0.02	2.16	1.63-2.81	26	262	134	5 (0.2)	21 (21.0)	79 (79.0)	2,895 (99.8)	84
												*Min*^§ ^= *84*

Average simulation results: we show the results from the joint model on one case of simulated data for independent experiments and six cases of simulated data for two associated experiments. The simulation scenario consists of four groups of genes: differentially expressed DE in both experiments, differentially expressed in only one experiment (DE1 and DE2 respectively), and differentially expressed in neither experiment. For the Independence case, the number of genes differentially expressed in both experiments was set to 0. We present two decision rules: the threshold associated with the maximum *R*(*q*) is *q*_*max *_and the threshold associated with the *R*(*q*) ≥ 2 is *q*_2 _(called 'double' in the table). We define *q*_*max *_= arg max{Median(R(*q*) | **O**, *n*) over the set of values of *q *for which *CI*_95_(*q*) excludes 1} and *q*_2 _= max{over the set of values of *q *for which *CI*_95_(*q*) excludes 1 and *Median*(*R*(*q*) | **O**, *n*) ≥ 2}. We averaged the results over 50 repeats for each case. *In case of independence it is still possible to calculate he maximum of *R*(*q*), but it is not significant, so there is no associated list of common genes. ^†^All the CIs contain 1, so no genes are called in common; thus, there are no FP. ^‡^The signal to ratio is calculated as *E*(*Ga*(*shape*, 1/*scale*))/(*r*_1_/2 + *r*_2_/2). ^§^Minimum global error (observed). ^¶^There is no ratio larger than 2 and only the maximum rule has been reported.

Scenario I mimics a realistic situation where the two experiments have different degrees of differential expression and consequently quite different list sizes at any given significance level. It supposes that the list of genes is divided into four groups: genes differentially expressed in both experiments, genes differentially expressed in only one of the two experiments, and genes differentially expressed in neither experiment. The first group identifies the 'true positive genes' that we want to detect by our method. The remaining groups act like additional noise to make the set up more realistic. We also define a different scenario (scenario II) to mimic a situation where the two experiments have similar size of differential expression. It only supposes the genes are divided into two groups: differentially expressed genes in both experiments and differentially expressed genes in no experiment. We describe the simulation set up in detail in Materials and methods.

In both scenarios, structure A refers to experiments where there would be a large proportion of genes in common relative to the total number of differentially expressed genes. Case A1 is characterized by a large true difference between conditions and a small experiment-specific error, giving an average signal-to-noise ratio of 9.6. Our first rule returns a ratio *T*(*q*_max_) = 2.61 that is associated with *q*_max _= 0.01. In this case the average number of genes in the common list associated with the max ratio is *O*_11_(*q*_max_) = 619, while that expected is 975×7303000=237
 MathType@MTEF@5@5@+=feaafiart1ev1aaatCvAUfeBSjuyZL2yd9gzLbvyNv2Caerbhv2BYDwAHbqedmvETj2BSbqee0evGueE0jxyaibaiKI8=vI8tuQ8FMI8Gi=hEeeu0xXdbba9frFj0=OqFfea0dXdd9vqai=hGuQ8kuc9pgc9s8qqaq=dirpe0xb9q8qiLsFr0=vr0=vr0dc8meaabaqaciGacaGaaeqabaqadeqadaaakeaadaWcaaqaaiaaiMdacaaI3aGaaGynaiabgEna0kaaiEdacaaIZaGaaGimaaqaaiaaiodacaaIWaGaaGimaiaaicdaaaGaeyypa0JaaGOmaiaaiodacaaI3aaaaa@3FFA@ and the permutation based test returns a significant Monte Carlo *p *value ≤ 0.001. The Bayesian ratio *R*(*q*_max_) is slightly smaller than *T*(*q*_max_); accounting for variability in the Bayesian model results in wide CIs for small *p *values as previously pointed out. Our methodology gives excellent results in this case, with the sum of false positives and false negatives equal to 89, while the FDR is 0.006 and the FNR is 0.036. Moving from *q*_max _to *q*_2_, the number of genes called in common by this procedure is 676, which is very close to the true number of common genes set in the simulation (700). The number of false positives is larger than the one corresponding to *q*_max_, but still quite small, whilst the number of false negatives decreases appreciably, so that the global error reaches its minimum value (83). Note that both *q*_max _and *q*_2 _generate a far smaller global error than Hwang *et al*.'s procedure (Table 3).

Moving to case A2, the noise associated with the experiment increases and the true differences between conditions are smaller. This results in fewer genes called in common and a corresponding increase in the global error. Nevertheless, all the cases present the same trend: *q*_max _is associated with the synthesized list having the smallest number of false positives and the list given by *q*_2 _is close to the one with the smallest global error. Moreover, for both cut-offs our methodology consistently leads to smaller errors than that of Hwang.

Simulations under structure B and C mimic cases where there is a smaller proportion of genes in common relative to the total number of differentially expressed genes. For cases B1 and C1 the noise is very small and the true difference between conditions is large; cases B2 and C2 are characterized by a smaller true difference and a higher noise. The pattern remains the same in cases A1 and A2: the list associated with *q*_max _shows the smallest number of false positives, while the one associated with *q*_2 _is very close to the minimum global error. Again our rules show a far smaller global error that those of Hwang. Note that for cases B1 and C1, there is no *q*_2 _and *q*_max _is associated with the smallest global error. Additional simulations are presented in Tables 1 and 2 of Additional data file 1.

Scenario II shows a similar trend confirming that our method also works well in a different experimental framework. We still find very few false positives with both rules *q*_max _and *q*_2_. On the other hand, the sensitivity is generally higher than in scenario I for both rules, hence the global error is smaller. This results in a better performance of the maximum *q*_max_: it shows no false positive in all the cases of this scenario and since the false negatives are generally fewer, its global error is quite small and, in some cases, smaller than the one for *q*_2_. Hwang *et al*.'s method shows an improvement in terms of false positives with respect to scenario I, while the false negatives remain quite the same. This is to be expected because, in this scenario, the intersection and the union of differentially expressed genes are identical. Nevertheless, our method also performs better in most of the cases in this scenario, with the exception of case A2, where our global error is 509 for the *q*_2 _rule while Hwang *et al*.'s is 450. However, we still halve the number of false positives. See Tables 3 and 4 of Additional data file 1 for the results under scenario II.

### Common features related to ventilation-induced lung injury

We applied our methods to lists of *p *values for 2,769 mouse and rat orthologs deriving from a study investigating the deleterious effects of mechanical ventilation on lung gene expression through a model of mechanical ventilation-induced lung injury (VILI; see Materials and methods for details of this study). Results from the joint model are summarized in Table 5 and the plots are presented in Figure 2 of Additional data file 1. The conditional model returns nearly identical results. Due to the large variability there is no threshold associated with a *R*(*q*) ≥ 2, so we present the results related to *q*_max_. The number of differentially expressed genes common to both species is estimated as 97, which corresponds to 63 orthologs (note that each probeset of one species can be associated with several probesets of the other). These are presented in Additional data file 1, which shows the number of ortholog pairs in common out of the number of ortholog pairs measured.

**Table 5:** Results from the VILI experiment

Joint Bayesian model						Hwang *et al*.'s method	
	
*q*_*max*_	*R*(*q*_*max*_)	*O*_11_	*O*_1+_	*O*_+1_	*CI*_95_	DE	nonDE
0.01	1.43	97	393	886	1.13-1.75	1,425	3,734

The number of genes in common is 97, which corresponds to 63 orthologs. The conditional model shows the same results (not reported). The procedure indicates clearly a significant association between the two lists. Hwang *et al*.'s method calls 1,425 genes as differentially expressed (DE). All the genes reported by our method are included in their list.

We compared our results to those obtained applying Hwang *et al*.'s method, also presented in Table 5. The latter picked 1,425 globally differentially expressed genes using the non-parametric rule. The 97 genes in common found by our method are included in their list, which is not surprising since ours focuses on the intersection of the two lists of *p *values, while theirs tests their union.

**Figure 4:**
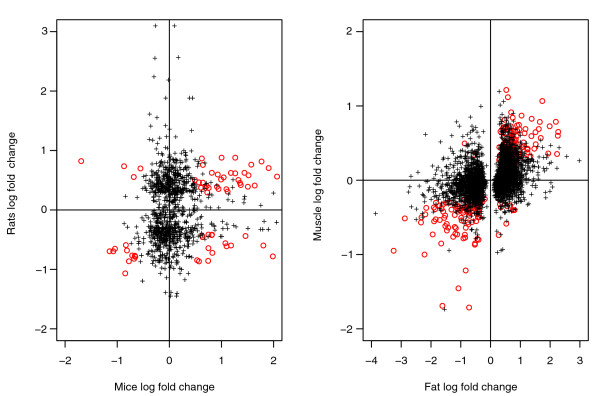
Log fold change (natural log) for the VILI experiment (left) and high-fat diet experiment (right). The left plot shows the log fold changes for mice versus rat averaged over the two replicates for each species. The right plot shows the log fold changes for fat versus muscle averaged over the three and four replicates for each species. The circles correspond to the genes highlighted by our analysis and by the method of Hwang *et al*.; they are characterized by a large log fold change for both the species. The correlation of the two fold changes for this group is 0.4 (VILI experiment) and 0.8 (high-fat diet experiment). The crosses correspond to the genes highlighted only by Hwang *et al*.'s analysis; they are characterized by a large log fold change for one species and a small fold change for the other one. The correlation of the two fold changes for this group is 0.06 (VILI experiment) and 0.36 (high-fat diet experiment).

This difference is highlighted in Figure 4 (left), which plots mice fold change versus rats fold change on the natural logarithmic scale: it is apparent that genes highlighted by Hwang *et al*.'s method but not by ours (+) have log fold change close to 0 for one of the species, while the genes highlighted by both the methodologies (*o*) present large fold changes for both the species. The correlation between the fold changes measured in the two experiments is 0.4 for the 97 orthologs returned by our procedure and 0.06 for the other 1,328 genes picked up only by Hwang *et al*.'s method, confirming how our methodology focuses attention on the genes differentially expressed in both experiments.

We used fatiGO [12] to annotate the common set of orthologs found by our analysis: 24 genes are involved in one or more pathways described in the Kyoto Encyclopedia of Genes and Genomes (KEGG), 42 are annotated at the third level of the Gene Ontology (GO) as part of biological processes, 41 belong to molecular functions and 36 to cellular components. See Additional data file 2 for the complete list of GO categories and KEGG pathways.

Out of the biological processes, the most represented are related to the integrated function of a cell ('cellular physiological process', 'metabolism', 'regulation of cellular process', 'regulation of physiological process'), showing between 38 and 15 orthologs in common. In addition, there are some other interesting processes related to responses of the body to stress and external or endogenous stimulus; these can be related to the effect of mechanical ventilation, which acts as an external stimulus and also causes stress on cells.

From the KEGG pathways, we focus attention on the two most represented categories: the 'MAPK signaling activity' and the 'Cytokine-cytokine receptor interaction'. Six of the orthologs found to be significant are involved in the first (*Fgfr1*, *Gadd45a*, *Hspa8*, *Hspa1a*, *Il1b*, *Il1r*_2_). The involvement of this pathway is again suggestive of how mechanical ventilation acts as an external stimulus, causing inflammation and eventually also apoptosis. The gene encoding fibroblast growth factor receptor 1 (*Fgfr1*) seems particularly interesting; it belongs to the GO category 'GO:0030324', related to lung development.

Five of the orthologs found to be significant by our methodology belong to the 'Cytokine-cytokine receptor interaction' category (*IL6*, *Il1b*, *Il1r*_2_, *Ccl2*, *Kit*). This again suggests an involvement of immune response in VILI for both species.

These results clearly show that our procedure gives a coherent list of genes that are differentially expressed in both species and is consequently a powerful procedure for finding common pathways of interest.

### Common features related to high-fat diet

**Figure 5:**
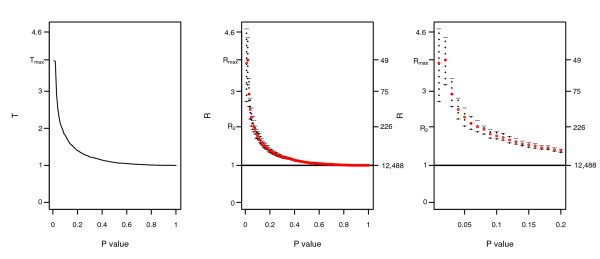
Results from the high-fat diet experiment. The left plot shows the distribution of *T*(*q*) and the center one shows the distribution of *R*(*q*) with Bayesian credibility intervals at 95%. *q*_*max *_for the conditional model is 0.01 and returns 20 genes in the common list, whilst for the joint model it is 0.02 and returns 49 common genes. On the other hand, *q*_2 _= 0.07 and the number of genes in common is 226. The left plot is a blow-up of the Bayesian model results, to better visualize the trend for *p *values between 0 and 0.2. The number of genes in common for each ratio is reported on the right axis of each plot.

We applied our methodology to the list of 12,488 genes from original experiments evaluating the effects of high fat diet versus normal fat diet in muscle and adipose tissue of two strains of mice (see Materials and methods for details of this study). The results from the Bayesian model are reported in Table 6 and Figure 5 and are confirmed by the conditional model (data not shown). We include in the table both the decision rules, *q*_max _and *q*_2_. The ratio *R*(*q*_max_) associated with the first decision rule is 3.84 with a *CI*_95 _of 3.17-4.44. The number of genes in common is 49. On the other hand, the ratio associated with the other decision rule, *R*(*q*_2_) is 2.04 and it returns a *CI*_95 _of 1.90-2.21. In this case the number of common genes is 226.

**Table 6:** Results from high-fat diet experiment

	Joint Bayesian model						Hwang *et al*.'s method	
		
Rules	*q*	*R*(*q*)	*O*_11_	*O*_1+_	*O*_+1_	*CI*_95_	DE	nonDE
Max	0.02	3.83	49	1,893	85	2.72-4.68	3,746	8,742
Double	0.07	2.04	226	3,059	452	1.90-2.21	3,746	8,742

The joint model returns *R*(*q*_*max*_) = 3.83 with an associated credibility interval [3.17-4.44]. The conditional model shows the same results (data not reported). The *R*(*q*_2_) is 2.04 and the CI is 1.90-2.21. The procedure indicates clearly a significant association between the two lists. Hwang *et al*.'s method calls 3,746 genes as differentially expressed (DE). All the genes called by our method are included in their list.

As was already ascertained with the VILI datasets, the list of genes in common found using our methodology is contained in that found using Hwang *et al*.'s methodology. The latter declared 3,746 genes as differentially expressed (DE; Table 6) and, looking at the fold changes of this (Figure 4, right), confirms again how our methodology finds the intersection between experiments, while that of Hwang *et al*. tests their union. The correlation of the two lists of fold changes for the genes declared by our methodology and Hwang *et al*.'s is 0.8, while that for the genes called only by Hwang *et al*.'s procedure is 0.36. A union of differentially expressed genes from muscle and fat inevitably contains many tissue-specific responses, whereas the intersection has the potential to reveal common underlying tissue adaptation or systemic responses to a high fat diet switch.

The size of the differentially expressed genes for each cut-off *q *is quite different for the two tissues. This is an example of the simulated scenario I, where we clearly also expect to have genes differentially expressed only in one tissue. For this reason, we focused particular attention on the rule *q*_2_, which showed a smaller global error under scenario I. Again, we used fatiGO [12] to annotate the 226 genes found by the *q*_2 _rule: 128 are involved in the GO category 'Biological processes', 107 in 'Molecular function' and 116 in 'Cellular components'; 42 belong to at least one KEGG pathway. The complete annotation is reported in Additional data file 3.

Of special note in the KEGG pathways are several classes of genes involved in inflammation and glucose metabolism. It is well known that insulin resistance in mammals is associated with chronic inflammation in adipose tissue [13]. Indeed, the top KEGG category in our analysis is 'Cytokine-cytokine receptor interaction', and includes the genes *Ccl2 *and *Tnfrsf1b*. The *Ccl2 *gene encodes a C-X-C family cytokine that is a ligand for the receptor *Ccr2*, a key mediator of diet-induced obesity and insulin resistance [14]. *Tnfrsf1b *encodes a receptor for tumor necrosis factor, an inflammatory cytokine that is well established to be an inducer of insulin resistance in adipose tissue [15,16]. It is particularly interesting, therefore, to see that inflammatory genes are also perturbed in muscle by the switch to a high-fat diet, suggesting that similar molecular events are brought about in these two tissues in response to the change in diet. Another interesting category at the top of the list is 'Neuroactive ligand receptor interaction', which includes *Leptin*, *GHR *and *NR3C1*. Leptin and growth hormone receptor (*GHR*) are known in the literature to be associated with obesity and a high-fat diet in several tissues [17]; nuclear receptor subfamily 3, group C, member 1 (*NR3C1*) is a ligand-activated transcription factor that interacts with high affinity with cortisol and other glucocorticoids. It is involved in response to stress and in the suppression of the immune system. Activation of expression of *NR3C1 *within the liver may contribute to the development of type 2 diabetes in mice [18] and it has a role in liver glucose metabolism during fasting and in diabetic mice [19]. It would be very interesting to further investigate if its role is maintained in other tissues besides fat and muscle, as suggested by our analysis.

The proposed method highlights some interesting GO categories as 'Mitochondrial function' (Cellular component: Mitochondrion) and 'Oxidative reactions' (Molecular functions category) are highlighted. Oxidative stress in adipose tissue and adipocytes is associated with the development of insulin resistance [16,20], although the mechanisms underlying this are not understood. Furthermore, there is impaired insulin-stimulated mitochondrial energy production in muscle of type 2 diabetic patients [21].

That these processes are identified using our method of analyzing differentially expressed genes from diet-induced obesity shows the strength of our approach. Using a concise, well calibrated list, features of known biological interest as well as novel aspects (for example, the KEGG pathway 'Neuroactive ligand-receptor interactions' and particularly the gene *NR3C1*) can be identified for further investigation.

### Modeling three way contingency tables

The methodology presented can be generalized to more than two experiments. Suppose we want to compare *m *experiments through *m *lists of *p *values. The associated contingency table will have dimension 2^*m*^.

In an *m*-way table, different hypotheses of independence can be considered [22]. We refer here to mutual independence as a direct extension of what we presented for a 2 × 2 table and discuss other types of independence in the Discussion.

Considering three experiments and using a similar notation to that previously introduced, for each threshold *q *we define the experiments mutually independent if:

O111(q)=O1++′O+1+′O++1n2
 MathType@MTEF@5@5@+=feaafiart1ev1aaatCvAUfeBSjuyZL2yd9gzLbvyNv2Caerbhv2BYDwAHbqedmvETj2BSbqee0evGueE0jxyaibaiKI8=vI8tuQ8FMI8Gi=hEeeu0xXdbba9frFj0=OqFfea0dXdd9vqai=hGuQ8kuc9pgc9s8qqaq=dirpe0xb9q8qiLsFr0=vr0=vr0dc8meaabaqaciGacaGaaeqabaqadeqadaaakeaacaWGpbWaaSbaaSqaaiaaigdacaaIXaGaaGymaaqabaGccaGGOaGaamyCaiaacMcacqGH9aqpdaWcaaqaaiaad+eadaqhaaWcbaGaaGymaiabgUcaRiabgUcaRaqaaOGamaiYgkdiIcaaieaacaWFpbWaa0baaSqaaiabgUcaRiaaigdacqGHRaWkaeaakiadaISHYaIOaaGaa83tamaaBaaaleaacqGHRaWkcqGHRaWkcaaIXaaabeaaaOqaaiaad6gadaahaaWcbeqaaiaaikdaaaaaaaaa@4BE7@

The statistic *T*(*q*) is generalized to:

T(q)=O111(q)O1++(q)×O+1+(q)×O++1(q)n2
 MathType@MTEF@5@5@+=feaafiart1ev1aaatCvAUfeBSjuyZL2yd9gzLbvyNv2Caerbhv2BYDwAHbqedmvETj2BSbqee0evGueE0jxyaibaiKI8=vI8tuQ8FMI8Gi=hEeeu0xXdbba9frFj0=OqFfea0dXdd9vqai=hGuQ8kuc9pgc9s8qqaq=dirpe0xb9q8qiLsFr0=vr0=vr0dc8meaabaqaciGacaGaaeqabaqadeqadaaakeaacaWGubGaaiikaiaadghacaGGPaGaeyypa0ZaaSaaaeaacaWGpbWaaSbaaSqaaiaaigdacaaIXaGaaGymaaqabaGccaGGOaGaamyCaiaacMcaaeaadaWcaaqaaiaad+eadaWgaaWcbaGaaGymaiabgUcaRiabgUcaRaqabaGccaGGOaGaamyCaiaacMcacqGHxdaTcaWGpbWaaSbaaSqaaiabgUcaRiaaigdacqGHRaWkaeqaaOGaaiikaiaadghacaGGPaGaey41aqRaam4tamaaBaaaleaacqGHRaWkcqGHRaWkcaaIXaaabeaakiaacIcacaWGXbGaaiykaaqaaiaad6gadaahaaWcbeqaaiaaikdaaaaaaaaaaaa@5497@

where *O*_1++_, *O*_+1+_, *O*_++1 _are again the marginal number of differentially expressed genes in each of the three experiments. It is known from the literature [23] that each cell of a contingency table conditional on the strata margins follows a hypergeometric distribution. Hence:

*O*_111_(*q*) ~ *Hyper*(*O*_1++_(*θ*), *O*_+11_(*θ*), *ν*).

and, as previously pointed out, *T*(*q*) is proportional to a hypergeometric. Thus, the permutation based test can be used again to evaluate the significance of *T*(*q*_*max*_).

Releasing the conditioning on the margins, the sampling schema is multinomial, as presented in equation 3, but with 2^*m *^- 1 parameters and the statistic of interest is a direct extension of equation 4. The decision rules defined in equations 6 and 7 can be applied to *R*(*q*).

To show that this extended procedure works well for synthesizing three lists of *p *values, we enlarged our simulations to include a case of three experiments following scenarios I, B and C already presented for two experiments.

Performing 50 simulations for each scenario, we found consistent results. *q*_max _picks few genes and it is very conservative. It declares no false positives but, as expected, many false negatives. On the other hand, *q*_2 _shows a larger list than *q*_max_, but is characterized by still few false positives and a global error close to the minimum observed (see Table 5 of Additional data file 1 for the results).

## Discussion

Intersecting lists of differentially expressed features is a natural way to synthesize experiments, but calls for a statistical procedure to choose the cut-off on the ranked lists that is best for balancing specificity and sensitivity.

We have demonstrated how our methodology gives statistically meaningful cut-offs and how it has the benefit of not requiring the original data, but only a probability measure of differential expression for each list, as a *p *value. For this reason, it can easily be applied to many types of experiments, including those carried out on different platforms or on different species. Moreover, the comparison can be performed at the gene level or at the function level and uses the type of classification function that is most relevant. In the latter case, *p *values have to be related to each function instead of each gene, using for instance the methodology for global testing of biological functions described in Goeman *et al*. [24].

The list of *p *values is not the only possible strategy for ranking the genes; on a probability scale, posterior probability for a gene to be differentially expressed can also be used [25], being aware that, in this case, the ranking should be inverted so that large posterior probabilities correspond to genes most differentially expressed. Outside the probability scale, the fold change could also be used as a ranking variable. However, while the range based on the probability scale is easily defined, that of the fold change will vary for each experiment and researchers should define a global range of values that is sensible for synthesizing all the comparisons.

We have simulated two scenarios that reflect different experimental setups. Scenario I supposes that in the two experimental conditions under study, there are some condition specific genes, differentially expressed only in one of the two experiments, as well as common genes. On the other hand, scenario II supposes that all the genes are either differentially expressed in both experiments or differentially expressed in neither. Both scenarios are plausible, but we think that the first one is more likely to occur when analyzing experimental data. Indeed, in both our case studies, there was a strong indication towards scenario I, with different sized lists for each species or each tissue (see in Tables 5 and 6 the differences between *O*_1+_(*q*) and *O*_+1_(*q*)). It is thus particularly interesting to focus attention on the common genes, because it returns the ones conserved between species (VILI experiment) or potentially responsible for some biological mechanisms that remain the same between different tissues (high-fat diet experiment).

Both the conditional and joint models we propose are based on the simplifying assumption of independence within the set of genes under study. This assumption allows one to define the underlying distribution as multinomial, but is clearly an oversimplification in the context of genomic data. We evaluated through an additional set of simulations described in Materials and methods how the results of our procedures would be affected if the features in each experiment were correlated. We found that analyzing a correlated set of genes with our method tends to inflate the estimates of the ratio under both the conditional and joint models for small *p *values. Hence, the threshold *q*_2 _is larger than that for the simulation of an independent set of genes (0.04 versus 0.02). Nevertheless, in terms of false positives, false negatives and global misclassification error, we find that performance is similar to when the genes are not correlated (see Table 6 and Figure 3 of Additional data file 1).

These results show that even though the independence assumption is unrealistic, it does not substantially alter the performance of our method. To reduce the dependence, a possible extension of our method would be to consider groups of differentially expressed features that are linked through common pathways, for example, and to test whether the same groups are commonly perturbed across different experiments.

In the previous section, we also showed how we can extend our method to more than two experiments, focusing attention on three lists, but we stress that our methodology is readily extendable to more than three experiments. Since the marginal distribution of a multi-hypergeometric is again hypergeometric, the calculations are simplified and the computing time does not increase exponentially from the two lists comparison case. Another convenient feature of our framework is that it can be applied to evaluate a variety of independence models for more than two experiments. We focused attention on mutual independence, but hypotheses of conditional independence or joint independence [22] can also be considered. The definitions of *T*(*q*) and *R*(*q*) have to be modified accordingly, but the methodology can be applied as it is. Moreover, the interest can be focused on negative association as well, which is on the *O*_1+_(*q*) - *O*_11_(*q*) and *O*_+1_(*q*) - *O*_11_(*q*) cells in the 2 × 2 table, corresponding to clearly specific features in each experiment that are not found under the other conditions.

We have presented two alternative rules to select the list of interest: the first is associated with the maximum ratio *R*(*q*), which quantifies the largest deviation from the independence. It is very specific but rather conservative and tends to select small lists. To achieve larger and balanced lists we have proposed a second rule based on a ratio *R*(*q*) ≥ 2 and have shown that this leads to the smallest observed global misclassification error (FP + FN). The comparison to Hwang *et al*.'s method has pointed out that our two rules perform better in terms of global error in a variety of realistic simulated scenarios. As a general comment, we suggest that the pattern of *R*(*q*) ratio*s *and associated significant credibility intervals are also discussed with the experimentalists, who can select between *q*_max _and *q*_2 _for the threshold most appropriate to their experimental context in terms of the relative weights of specificity and sensitivity.

## Conclusion

We have presented a simple methodology to synthesize several experiments with the aim of finding a statistically meaningful list of features that are perturbed in both (all) experiments and demonstrate that our procedures have excellent specificity and good sensitivity. They are applicable to a wide range of experiments and comparisons. They provide experimentalists with powerful exploratory tools that can help select a list of features of interest for further biological investigation, as demonstrated by our analysis of two real experimental datasets.

## Materials and methods

### Simulated data

To assess the performance of our methodology we use batches of simulation. We follow the simulation set up described in [2], so that comparison between the two approaches is easier. Considering two experiments (*k *= 1,2), each of them with two conditions, and *n *genes, for each gene we simulate a true difference between the conditions δ_*g*_, drawn from a gamma distribution with random sign. The true difference δ_*g *_is 0 if the gene is not differentially expressed. We then add a normal random noise, *r*_*k*_*ε*_*gk*_, where *r*_*k *_is the experiment specific component and *ε*_*gk *_is drawn from a standard Gaussian distribution and is experiment and gene specific. We set up two scenarios. In the first, which we call scenario I, we divided the *n *genes into four groups: genes differentially expressed in both experiments, genes differentially expressed only in the first experiment, genes differentially expressed only in the second experiment and genes differentially expressed in neither experiment. In the second scenario, called scenario II, we divided the genes into only two groups: genes differentially expressed in both experiments and genes differentially expressed in neither experiment. Scenario II is thus a particular case of scenario I, which assumes strong communality between the two experiments. When the genes are differentially expressed in both experiments, they share the same δ_*g *_and the only difference between them is given by the random components:

*T*_*g*1 _= *δ*_*g *_+ *r*_1_·*ε*_*g*1_

*T*_*g*2 _= *δ*_*g *_+ *r*_2_·*ε*_*g*2_

where *T *stands for the fold change on the logarithmic scale. This group represents the 'true positive genes' (that is, truly differentially expressed in both experiments) that we are interested in finding using our method. In scenario I, the two groups of genes differentially expressed only in one of the two experiments act like additional noise and make the simulation more biologically realistic. Together with the genes not differentially expressed they constitute the 'negative genes' in this setup, that is, genes that should not be listed if the procedure correctly identifies the intersection.

Then, as described in [2], a two tailed *t*-test is performed for each *T*_*gk *_and a *p *value is generated as:

pgk=2Ncdf(−|Tgkrk|)
 MathType@MTEF@5@5@+=feaafiart1ev1aaatCvAUfeBSjuyZL2yd9gzLbvyNv2Caerbhv2BYDwAHbqedmvETj2BSbqee0evGueE0jxyaibaiKI8=vI8tuQ8FMI8Gi=hEeeu0xXdbba9frFj0=OqFfea0dXdd9vqai=hGuQ8kuc9pgc9s8qqaq=dirpe0xb9q8qiLsFr0=vr0=vr0dc8meaabaqaciGacaGaaeqabaqadeqadaaakeaacaWGWbWaaSbaaSqaaiaadEgacaWGRbaabeaakiabg2da9iaaikdacaWGobWaaSbaaSqaaiaadogacaWGKbGaamOzaaqabaGcdaqadaqaaiabgkHiTiaacYhadaWcaaqaaiaadsfadaWgaaWcbaGaam4zaiaadUgaaeqaaaGcbaGaamOCamaaBaaaleaacaWGRbaabeaaaaGccaGG8baacaGLOaGaayzkaaaaaa@4549@

Both our method and that of Hwang *et al*. use the lists of *p *values as a starting point, so we implemented both procedures and compared the results in terms of false positives and false negatives. To be precise, we call a gene a false positive (FP) if it is not differentially expressed in both the experiments but is called in common by the methodology, and we call a gene a false negative (FN) if it is differentially expressed in both the experiments but is not declared as in common by the methodology. We also report the complementary quantities of true positives (TP) and true negatives (TN) that characterize the sensitivity and the specificity, respectively, of a rule. For each scenario, we defined three structures, differing in the size of intersection (Table 7).

**Table 7:** Simulation schema

	Common genes (DE in both experiments)	DE only in first experiment	DE only in second experiment	Non DE
A	700	300	100	1,900
B	200	500	300	2,000
C	100	400	300	2,200

DE, differentially expressed.

Within each structure we further varied the value of the true differences and the level of noise for each experiment, giving cases 1 and 2. In case 1, the true differences δ_*g *_are drawn from a *Ga*(2.5,0.4) and the level of noise is very small (*r*_1 _= 0.5 and *r*_2 _= 0.8). In case 2, the true differences δ_*g *_are drawn from a *Ga*(2,0.5) and the level of noise is larger (*r*_1 _= 2 and *r*_2 _= 3). We also simulated a null scenario where the experiments are independent and do not share values of δ_*g*_, with 1,000 genes differentially expressed only in the first experiment, 800 differentially expressed only in the second and 1,200 not differentially expressed. For scenario II we replicated the same structure and cases but with only two groups of genes. For every case, we performed 50 simulations and averaged the results for both the methods. Additional simulation results with different levels of differential expression and noise can be found in Tables 1 and 2 of Additional data file 1.

### Simulated data for three lists

We simulated data from three experiments adapting case 2 of scenario I, structures B and C. The true differences δ_*g *_are drawn from a *Ga*(2,0.5) and the experiment specific noises are *r*_1 _= 2, *r*_2 _= 2.5, *r*_3 _= 3. For structure B, we considered 200 genes in common, out of 700 differentially expressed in the first experiment, 600 differentially expressed in the second experiment and 500 differentially expressed in the third experiment. For structure C, we set 100 genes in common, out of 500 differentially expressed in the first experiment, 400 differentially expressed in the second experiment and 300 differentially expressed in the third experiment.

### Simulated data for a correlated set of genes

We simulated log gene expression data for 3,000 genes for two experiments with two classes. For each experiment the log gene expressions were drawn from a multivariate normal distribution and we imposed a correlation matrix adapted from experimental data we have been analyzing (BAIR project [26]). For the first experiment, the quartiles of the correlation coefficients are -0.86, -0.22, 0.01, 0.25, 0.85, while for the second experiment they are -0.96, -0.27, 0, 0.28, 0.97. The mean variance for the first experiment is 1.32 and for the second is 0.80. We divided the 3,000 genes into four groups following the setup described before in accordance with scenario I, structure A. For the differentially expressed genes the log expression of the first class was drawn from a multivariate normal with mean 12, while the log expression of the other class was drawn from a multivariate normal with mean equal to 5; for the not differentially expressed genes both the log expressions were drawn from a multivariate normal with mean 5. Out of the 3,000 simulated genes, the 700 in 'common' are simply differentially expressed in both experiments, but do not share a common differential effect.

We simulated four replicates for each condition in each experiment and used Cyber-T [27] to analyze the two experiments separately and to obtain the lists of *p *values. Cyber-T is a statistical program that can be conveniently used on high-dimensional array data for the identification of statistically significant differentially expressed features. It employs regularized *t*-tests based on an estimate of the variability among the measurements proposed by Baldi and Long [28]. The variance of each feature is calculated using a sliding window of genes with similar expression. The regularized *t*-test returns a *p *value for each feature. We used 101 as the sliding window and a 'confidence estimate value' for the Bayesian prior of 12.

As a point of comparison, we also simulated an identical scenario for a set of 3,000 uncorrelated genes (imposing 0 covariances for the multivariate normal). We performed 50 simulations and averaged the results for both scenarios (see Table 6 and Figure 3 of Additional data file 1).

### Publicly available dataset: synthesizing VILI between two species

We re-analyzed the data described by Ma *et al*. [29] that are available from the Gene Expression Omnibus [30]. The experiment was designed to investigate deleterious effects of mechanical ventilation on lung gene expression through a model of mechanical ventilation-induced lung injury (VILI). The experiment was conducted on two species of rodents, mice and rats, and is a good case study to evaluate whether our methodology can provide valuable insights for synthesizing multi-species experiments.

The data are available as .CEL files. There are two conditions (control and ventilation) and two replicates for each species. The eight arrays have been background corrected and normalized using the RMA function available through Bioconductor [31]. Since our methodology has the advantage of needing only a probability measure (for example, *p *value), we processed the dataset from the two species separately using Cyber-T and extracted the *p *values as input for the analysis. We used the recommended default parameters (a sliding window of 101 genes and a 'confidence estimate value' for the Bayesian prior of 6). We used the list of 2,769 orthologs for the two species from the original paper.

### Publicly available dataset: effect of high-fat diet versus normal fat diet in mice fat and muscle

We re-analyzed data from an experiment publicly available on the Diabetes Genome Anatomy Project website [32]. It has been designed to evaluate the effect of high fat diet versus normal fat diet in muscle and fat for two strains of mice (B6 and 129). We worked on the data related to the 129 mice strain. It is a good case study to evaluate whether our methodology works well for synthesizing results across different tissues.

The data are available as .CEL files. There are two conditions (normal-fat diet and high-fat diet) and two tissues (fat and muscle). The number of replicates is three for each of the two conditions in fat and four for each of the two conditions in muscle.

We analyzed the two tissues separately; we normalized each of them using RMA and applied Cyber-T, using default parameters (sliding window of 101 and a 'confidence estimate value' for the Bayesian prior of 9 for fat and 12 for muscle). We used the list of 12,488 genes on the *MG*_*U*_*74Av2 *chip.

## Additional data files

The following additional data are available with the online version of this paper. Additional data file 1 contains the results (tables and plots) for additional simulated cases under scenario I, for all the cases under scenario II, for correlated versus uncorrelated sets of genes and for the simulation of three lists. Additional data file 2 is a list of common genes for the VILI example with GO and KEGG annotations. Additional data file 3 is a list of common genes for the high-fat diet example with GO and KEGG annotations.

## Supplementary Material

Additional data file 1 Results (tables and plots) for additional simulated cases under scenario I, for all the cases under scenario II, for correlated versus uncorrelated sets of genes and for the simulation of three lists. Click here for file

Additional data file 2 Common genes for the VILI example with GO and KEGG annotations. Click here for file

Additional data file 3 Common genes for the high-fat diet example with GO and KEGG annotations. Click here for file
