# Supplementary material for: Statistical tools for synthesizing lists of differentially expressed features in related experiments
Source: Genome Biol. 2007 Apr 11;8(4):R54. doi: 10.1186/gb-2007-8-4-r54 (PMC1896017; doi:10.1186/gb-2007-8-4-r54)

Statistical tools for synthesizing lists of differentially expressed features in related experiments

Marta Blangiardo*[1] and Sylvia Richardson[1]

[1]Centre for Biostatistics, Imperial College. St. Mary's Campus, Norfolk Place London W2 1PG, UK

Email: Marta Blangiardo*- m.blangiardo@imperial.ac.uk;

*Corresponding author

Additional Results

Table 1: Performance on simulated data

Average simulation results: we show the results from the joint model on 3 cases of simulated data under Scenario I for two associated experiments. We present two decision rules: q_{max} and q_2. We define $q_{max} = \arg\max\{Median(R(q) \mid \mathbf{O}, n)$ over the set of values of q for which $CI_{95}(q)$ excludes 1$\}$ and $q_2 = \max\{$ over the set of values of q for which $CI_{95}(q)$ excludes 1 and $Median(R(q) \mid \mathbf{O}, n) \geq 2\}$. We average the results over 50 repeats for each case.

**Scenario I: n=3000,common=700, DE1=1000, DE2=800**												
Parameters	Rules	q	R	$CI\%95$	O_{11}	O_{1+}	O_{+1}	FP (%)	TP (%)	FN (%)	TN (%)	Glob. Err.
Case A3	max	0.01	2.88	2.76-3.02	462	850	565	3 (0.1)	459 (65.6)	241 (34.4)	2297 (99.9)	244
Ga(2.5,0.4);	double	0.08	2.01	1.94-2.09	588	1067	823	41 (1.7)	547 (78.1)	153 (21.9)	2259 (98.3)	194
$r_1 = 1, r_2 = 1.5$												
Signal to noise												
ratio = 5[b]												$Min^c = 192$
Case A4	max	0.01	4.13	3.83-4.46	187	478	284	2 (0.1)	185 (86.4)	515 (73.6)	2298 (99.9)	517
Ga(2,0.5);	double	0.09	2.02	1.91-2.13	348	829	624	39 (1.7)	309 (13.4)	391 (55.8)	2261 (98.3)	430
$r_1 = 1.5, r_2 = 2$												
Signal to noise												
ratio = 2.3[b]												$Min^c = 428$
Case A5	max[d]	0.04	1.57	1.07-2.18	16	216	141	4 (0.2)	12 (1.7)	688 (98.3)	2296 (99.8)	692
Ga(1.5,0.6);												
$r_1 = 3, r_2 = 6$												
Signal to noise												
ratio = 0.7[b]												$Min^c = 692$

a All the CIs contain 1, so no genes are called in common. Hence there are no FP.

b The signal to ratio is calculated as $E(Ga(shape, 1/scale))/(r_1/2 + r_2/2)$

c Minimum global error (observed)

d There is no ratio larger than 2 and only the maximum rule has been reported

Table 2: Performance of Hwang's method on simulated data

Average simulation results: we present the results from Hwang's method on 3 cases of simulated data under Scenario I for associated experiments. We use the Fisher's weighted F defined as $F_g = -2\Sigma_{k=1}^{2} w_k ln(p_{gk})$ where w_k is the weight for the kth experiment and p_{gk} is the p-value for the gene g in the experiment k. We present the non parametric rule to select the differentially expressed genes, as suggested by the authors. The method is implemented in Matlab. In the last column we report the Global Error (FP+FN) of our procedure for q_2 (cfr. Table 1) for ease of comparison.

Scenario A: n=3000,common=700, DE1=1000, DE2=800								
	DE	n-DE	FP (%)	TP (%)	FN (%)	TN (%)	Glob.Err.	Glob.Err.$R(q_2)$
Case A3	1012	1988	386 (16.8)	626 (89.4)	74 (10.6)	1914 (83.2)	460	194
Case A4	700	2300	267 (11.6)	433 (61.8)	267 (38.2)	2033 (88.4)	534	430
Case A5	347	2653	208 (9.0)	139 (19.8)	561 (80.2)	2092 (91.0)	769	692[a]

a: there is no ratio larger than 2 so the maximum rule has been used in this case

Figure 1: Average results from a batch of 50 simulations: associated experiments, large noise and small true differences (Case A5)

The two associated experiments are simulated from Scenario A with true differences drawn from a Ga(1.5,0.67) and noise experiment specific of 3 and 6 respectively (signal to noise ratio = 0.7).
The upper plots show the distribution of $T(q)$ (left) and $R(q)$ with Bayesian Credibility Intervals at 95% (right). Starting from the right, $T(q)$ deviates from 1 around a p-value of 0.07 and reaches a peak of 1.7 at $q = 0.03$ and then decreases. $R(q)$ presents the same trend, but the CIs for small p-values show great variability of the lists and include 1. This is clearer in the blow-up plot for p-values between 0 and 0.2 (bottom, right): the credibility interval for q=0.03 includes 1, so it is not significant; the maximum over all the other intervals is attained at $q_{max} = 0.04$ and $R(q_{max}) = 1.57$ (recall that $q_{max} = \arg\max\{Median(R(q) \mid \mathbf{O}, n)$ over the set of values of q for which $CI_{95}(q)$ excludes $1\})$.

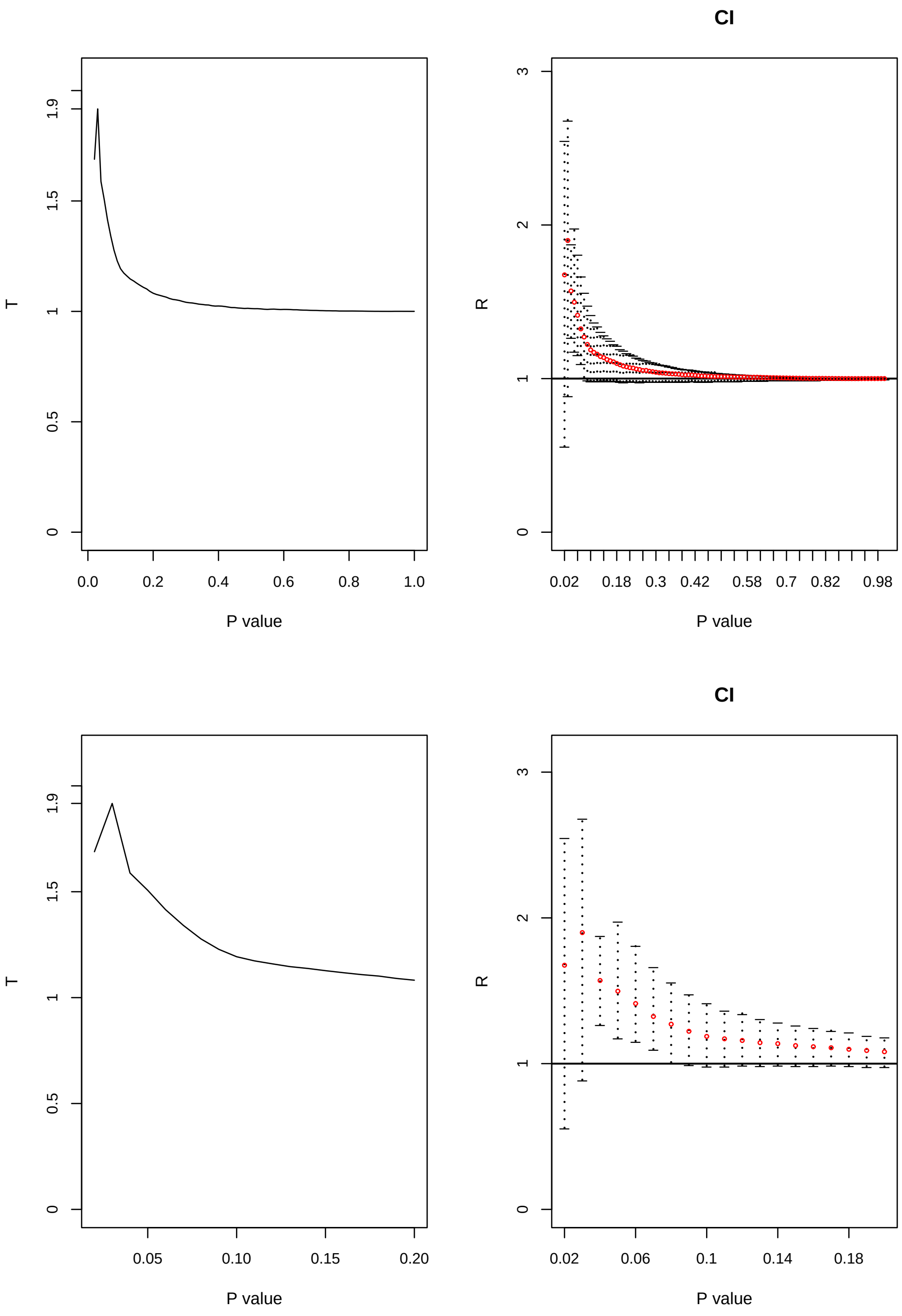
CI
CI
T
R
P value
P value
P value
P value
0.0
0.2
0.4
0.6
0.8
1.0
0
0.5
1
1.5
1.9
0
1
2
3
0.02
0.18
0.3
0.42
0.58
0.7
0.82
0.98
0.05
0.10
0.15
0.20
0.06
0.1
0.14
0.18

Table 3: Performance on simulated data for Scenario II

Average simulation results: from the joint model on six cases of simulated data for two associated experiments. The simulation scenario consists of 2 groups of genes: differentially expressed in both experiments and differentially expressed in neither experiment. We present two decision rules: the threshold associated with the maximum $R(q)$ is q_{max} and the threshold associated with the $R(q) \geq 2$ is q_2 (called 'double' in the table). We define $q_{max} = \arg\max\{Median(R(q) \mid \mathbf{O}, n)$ over the set of values of q for which $CI_{95}(q)$ excludes 1$\}$ and $q_2 = \max\{$ over the set of values of q for which $CI_{95}(q)$ excludes 1 and $Median(R(q) \mid \mathbf{O}, n) \geq 2\}$. We average the results over 50 repeats for each case.

A: n=3000,common=700													
Parameters	Rules	q	R	$CI95\%$	O_{11}	O_{1+}	O_{+1}	FP (%)	TP (%)	FN (%)	TN (%)	Glob.Err.
Case A1	max	0.01	4.12	3.93-4.33	622	699	650	0 (0.0)	622 (88.9)	78 (11.1)	2300 (100.0)	78
Signal to noise	double	0.14	2.11	2.04-2.19	711	994	1015	44 (1.9)	667 (95.3)	33 (4.7)	2256 (98.1)	77
ratio = 9.6[b]												*Min*[c] *= 57*
Case A2	max	0.01	8.11	7.31-9.06	101	246	150	0 (0.0)	101 (14.4)	599 (85.6)	2300 (100.0)	599
Signal to noise	double	0.13	2.03	1.92-2.13	271	671	598	40 (1.7)	231 (33.0)	469 (67.0)	2260 (98.3)	509
ratio = 1.6[b]												*Min*[c] *= 501*
B: n=3000,common=200												
Parameters	Rules	q	R	$CI95\%$	O_{11}	O_{1+}	O_{+1}	FP (%)	TP (%)	FN (%)	TN (%)	Glob.Err.
Case B1	max	0.01	11.16	10.26-12.60	179	226	212	0 (0.0)	179 (89.5)	21 (10.5)	2800 (100.0)	21
Signal to noise	double	0.14	2.08	1.93-2.22	229	589	562	41 (1.5)	188 (94.0)	12 (6.0)	2759 (98.5)	53
ratio = 9.6[b]												*Min*[c] *= 19*
Case B2	max	0.01	12.42	9.66-16.59	25	91	66	0 (0.0)	25 (12.5)	175 (87.5)	2800 (100.0)	175
Signal to noise	double	0.11	2.06	1.80-2.33	112	432	390	25 (0.9)	87 (43.5)	113 (56.5)	2775 (99.1)	138
ratio = 1.6[b]												*Min*[c] *= 138*
C: n=3000,common=100												
Parameters	Rules	q	R	$CI95\%$	O_{11}	O_{1+}	O_{+1}	FP (%)	TP (%)	FN (%)	TN (%)	Glob.Err.
Case C1	max	0.01	17.38	15.04-19.84	89	134	113	0 (0.0)	89 (89.0)	11 (11.0)	2900 (100.0)	11
Signal to noise	double	0.12	2.09	1.88-2.29	150	472	460	53 (1.8)	97 (97.0)	3 (3.0)	2847 (98.2)	56
ratio = 9.6[b]												*Min*[c] *= 7*
Case C2	max	0.01	11.99	7.60-18.15	11	61	44	0 (0.0)	11 (11.0)	89 (89.0)	2900 (100.0)	89
Signal to noise	double	0.05	2.02	1.37-2.47	22	187	177	3 (0.1)	19 (19.0)	81 (81.0)	2897 (99.9)	84
ratio = 1.6[b]												*Min*[c] *= 84*

b The signal to ratio is calculated as $E(Ga(shape, 1/scale))/(r_1/2 + r_2/2)$

c Minimum global error (observed)

Table 4: Performance of Hwang's method on simulated data for Scenario II

Average simulation results: we present the results from Hwang's method on the simulated data under Scenario II. We use the Fisher's weighted F defined as $F_g = -2\Sigma_{k=1}^{2} w_k ln(p_{gk})$ where w_k is the weight for the kth experiment and p_{gk} is the p-value for the gene g in the experiment k. We present the non parametric rule to select the differentially expressed genes, as suggested by the authors. The method is implemented in Matlab. In the last column we report the Global Error (FP+FN) of our procedure for q_2 (see Table 4) for ease of comparison.

A: n=3000,common=700								
	DE	nDE	FP (%)	TP (%)	FN (%)	TN (%)	GlobalError	GlobalErr $R(q_2)$
Case A1	753	2247	73 (3.2)	680 (97.2)	20 (2.8)	2227 (96.8)	92	77
Case A2	430	2570	90 (3.9)	340 (48.6)	360 (51.4)	2210 (96.1)	450	509
B: n=3000,common=200								
	DE	nDE	FP (%)	TP (%)	FN (%)	TN (%)	GlobalError	GlobalErr $R(q_2)$
Case B1	292	2708	98 (3.5)	194 (97.0)	6 (3.0)	2702 (96.5)	104	53
Case B2	240	2760	144 (5.1)	96 (48.0)	104 (52.0)	2656 (94.9)	249	138
C: n=3000,common=100								
	DE	nDE	FP (%)	TP (%)	FN (%)	TN (%)	GlobalError	GlobalErr $R(q_2)$
Case C1	203	2797	105 (3.6)	97 (97.0)	3 (3.0)	2795 (96.4)	108	56
Case C2	196	2804	149 (5.1)	47 (47.0)	53 (53.0)	2751 (94.9)	201	84

Figure 2: Results from the VILI experiment

The plots show the distribution of $T(q)$ (left) and $R(q)$ with the Bayesian Credibility Intervals at 95% (center). The right plot is a blow-up of the center one, showing the distribution for p-values between 0 and 0.2. The shape of $T(q)$ and $R(q)$ are similar, both exhibiting a monotone increase as p-values decrease. However, the Bayesian plot shows clearly how variability is taken into account, for small p-value the CIs becoming wider as the p-values decrease. The number of genes in common for each ratio is reported on the right axis of each plot.

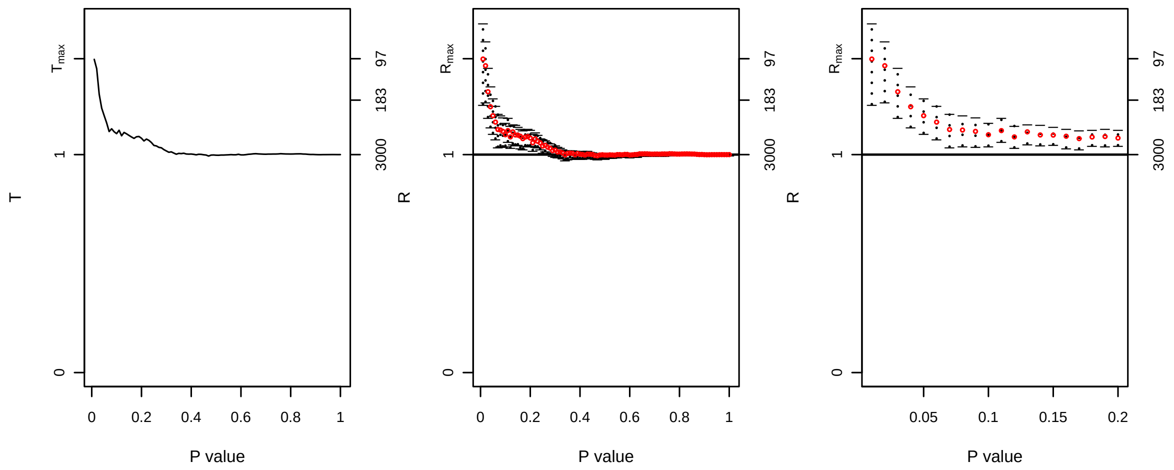

Table 5: Results of the simulation on three experiments

Average results from 50 simulation on three lists for the joint model: we report the three rules $R(q_{max})$ and $R(q_2)$. The first presents a small global error and no FP; the second has still few FP and the number of genes in common O_{111} is close to the true number set up in the simulation (200 for B1 and 100 for C1).

B: n=3000, common=200, DE1=700, DE2=600, DE3=500													
Parameters	Rules	q	R	95%CI	O_{111}	O_{1++}	O_{+1+}	O_{++1}	FP (%)	TP (%)	FN (%)	TN (%)	Glob.Err.
Case B1	max	0.01	35.26	24.54-47.92	18	187	170	143	0 (0.0)	18 (9.0)	182 (91.0)	2800 (100.0)	182
Signal to noise	double	0.17	2.04	1.76-2.34	92	736	761	727	32 (1.1)	60 (30.0)	140 (70.0)	2768 (98.9)	172
ratio = 1.6^b													Min^c=162
**C: n=3000, common=100, DE1=500, DE2=400, DE3=300**													
Parameters	Rules	q	R	95%CI	O_{111}	O_{1++}	O_{+1+}	O_{++1}	FP (%)	TP (%)	FN (%)	TN (%)	Glob.Err.
Case C1	max	0.01	45.13	26.20-70.55	9	188	120	80	0 (0.0)	9 (9)	91 (91)	2900 (100.0)	91
Signal to noise	double	0.15	2.11	1.71-2.56	52	682	605	536	16 (0.5)	36 (36.0)	64 (64.0)	2884 (99.5)	80
ratio = 1.6^b													Min^c=80

b The signal to ratio is calculated as $E(Ga(shape, 1/scale))/(r_1/3 + r_2/3 + r_3/3)$

c Minimum global error (observed)

Table 6: Comparing results on correlated and independent set of genes

Results of our methodology for 50 simulations on correlated sets of genes and on independent sets of genes. Treating a correlated sets of genes with our method inflates the estimates of the ratio under both conditional and joint model for small p-values. Hence, the threshold q_2 is larger than the one for the simulation of independent sets of genes (0.04 vs 0.02). In terms of specificity, sensitivity and global misclassification error we do not find large differences between the two cases.

**n=3000,common=700, DE1=1000, DE2=800; quartiles for correlation matrix in the first experiment: -0.86, -0.22, 0.01, 0.25, 0.85; quartiles for correlation matrix in the second experiment: -0.96, -0.27, 0.00, 0.28, 0.97**											
Rules	q	R	CI	O11	O1+	O+1	FP (%)	TP (%)	FN (%)	TN (%)	GlobalErr
max	0.01	2.68	2.12-3.24	45	292	173	2 (0.1)	43 (6.1)	657 (93.9)	2298 (99.9)	659
double	0.04	2.02	1.83-2.23	146	566	387	8 (0.3)	138 (19.7)	562 (80.3)	2292 (99.7)	570
											Min^c = *560*
**n=3000,common=700, DE1=1000, DE2=800; independence for the set of genes in the first experiment independence for the set of genes in the second experiment**											
Rules	q	R	CI	O11	O1+	O+1	FP (%)	TP (%)	FN (%)	TN (%)	GlobalErr
max	0.01	2.21	1.9-2.52	84	384	295	6 (0.3)	78 (11.2)	622 (88.8)	2294 (99.7)	628
double	0.02	2.01	1.8-2.22	128	498	384	14 (0.6)	113 (16.2)	587 (83.8)	2286 (99.4)	601
											Min^c = *554*

c Minimum global error (observed)

Figure 3: Credibility Intervals for correlated and independent set of genes

The plot shows the credibility intervals at 95% for $R(q)$, obtained using our joint model for the simulated scenario of correlated sets of genes (black) and the uncorrelated one (brown). It is clear that applying our methodology to correlated sets of genes results in an inflation of the estimates for small p-values. After 0.15 on the p-value scale the two sets of CIs overlap completely.

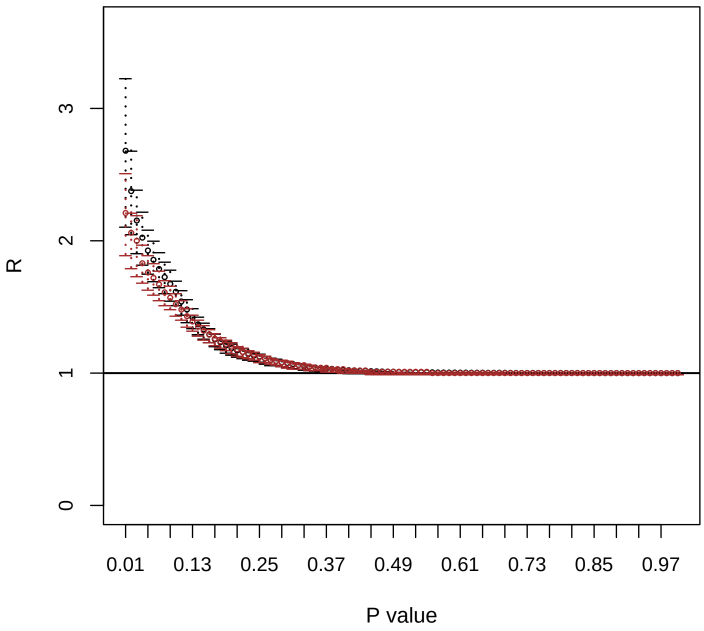

Supplement: Additional data file 1 — Results (tables and plots) for additional simulated cases under scenario I, for all the cases under scenario II, for correlated versus uncorrelated sets of genes and for the simulation of three lists. [file gb-2007-8-4-r54-S1.pdf]
